# A novel dual histone mark reader ZCWPW2 regulates meiotic recombination through lactylation and transcriptional regulation in humans and mice

**DOI:** 10.1093/nar/gkag049

**Published:** 2026-01-23

**Authors:** Tiechao Ruan, Jun Ma, Gan Shen, Xiang Wang, Yihong Yang, Liangchai Zhuo, Chuan Jiang, Guicheng Zhao, Yunchuan Tian, Shikun Zhao, Ruixi Zhou, Mohan Liu, Xinyao Tang, Yingteng Zhang, Chanjuan Zhao, Jincheng Zhang, Dingming Li, Xiaohui Jiang, Dezhi Mu, Lingbo Wang, Ying Shen

**Affiliations:** Department of Obstetrics/Gynecology, Key Laboratory of Birth Defects and Related Disease of Women and Children of MOE, West China Second University Hospital, Sichuan University, Chengdu 610041, China; Department of Obstetrics/Gynecology, Key Laboratory of Birth Defects and Related Disease of Women and Children of MOE, West China Second University Hospital, Sichuan University, Chengdu 610041, China; Department of Obstetrics/Gynecology, Key Laboratory of Birth Defects and Related Disease of Women and Children of MOE, West China Second University Hospital, Sichuan University, Chengdu 610041, China; Department of Obstetrics/Gynecology, Key Laboratory of Birth Defects and Related Disease of Women and Children of MOE, West China Second University Hospital, Sichuan University, Chengdu 610041, China; Department of Obstetrics/Gynecology, Key Laboratory of Birth Defects and Related Disease of Women and Children of MOE, West China Second University Hospital, Sichuan University, Chengdu 610041, China; Reproduction Medical Centre, West China Second University Hospital, Sichuan University, Chengdu 610041, China; Department of Obstetrics/Gynecology, Key Laboratory of Birth Defects and Related Disease of Women and Children of MOE, West China Second University Hospital, Sichuan University, Chengdu 610041, China; Department of Obstetrics/Gynecology, Key Laboratory of Birth Defects and Related Disease of Women and Children of MOE, West China Second University Hospital, Sichuan University, Chengdu 610041, China; Human Sperm Bank, Key Laboratory of Birth Defects and Related Diseases of Women and Children (Sichuan University), Ministry of Education, West China Second University Hospital, Sichuan University, Chengdu 610041, China; Department of Obstetrics/Gynecology, Key Laboratory of Birth Defects and Related Disease of Women and Children of MOE, West China Second University Hospital, Sichuan University, Chengdu 610041, China; Department of Obstetrics/Gynecology, Key Laboratory of Birth Defects and Related Disease of Women and Children of MOE, West China Second University Hospital, Sichuan University, Chengdu 610041, China; Department of Obstetrics/Gynecology, Key Laboratory of Birth Defects and Related Disease of Women and Children of MOE, West China Second University Hospital, Sichuan University, Chengdu 610041, China; Department of Obstetrics/Gynecology, Key Laboratory of Birth Defects and Related Disease of Women and Children of MOE, West China Second University Hospital, Sichuan University, Chengdu 610041, China; Department of Obstetrics/Gynecology, Key Laboratory of Birth Defects and Related Disease of Women and Children of MOE, West China Second University Hospital, Sichuan University, Chengdu 610041, China; Department of Obstetrics/Gynecology, Key Laboratory of Birth Defects and Related Disease of Women and Children of MOE, West China Second University Hospital, Sichuan University, Chengdu 610041, China; West China Institutes of Women and Children’s Health, West China Second University Hospital, Sichuan University. Key Laboratory of Birth Defects and Related Diseases of Women and Children, Ministry of Education. Si Chuan, Chengdu 610041, China; Department of Obstetrics/Gynecology, Key Laboratory of Birth Defects and Related Disease of Women and Children of MOE, West China Second University Hospital, Sichuan University, Chengdu 610041, China; Department of Obstetrics/Gynecology, Key Laboratory of Birth Defects and Related Disease of Women and Children of MOE, West China Second University Hospital, Sichuan University, Chengdu 610041, China; Human Sperm Bank, Key Laboratory of Birth Defects and Related Diseases of Women and Children (Sichuan University), Ministry of Education, West China Second University Hospital, Sichuan University, Chengdu 610041, China; Tianfu Jincheng Laboratory, City of Future Medicine, Chengdu 641400, China; Department of Obstetrics/Gynecology, Key Laboratory of Birth Defects and Related Disease of Women and Children of MOE, West China Second University Hospital, Sichuan University, Chengdu 610041, China; Shanghai Key Laboratory of Metabolic Remodeling and Health, Institute of Metabolism and Integrative Biology, Institute of Reproduction and Development, State Key Laboratory of Genetics and Development of Complex Phenotypes, Obstetrics and Gynecology Hospital, Fudan University, Shanghai 200433, China; Department of Obstetrics/Gynecology, Key Laboratory of Birth Defects and Related Disease of Women and Children of MOE, West China Second University Hospital, Sichuan University, Chengdu 610041, China; NHC Key Laboratory of Chronobiology, Sichuan University, Chengdu 610041, China

## Abstract

Meiotic recombination ensures accurate chromosome segregation and genetic diversity during gametogenesis, and its disruption leads to infertility. The dual histone methylation writer–reader system, in which PRDM9 deposits H3K4me3 and H3K36me3 marks at nucleosomes to define recombination hotspots and ZCWPW1 acts as a reader recognizing these marks, is essential for meiotic recombination. However, the regulatory mechanisms of this system remain unclear. Here, we showed that deficiency of ZCWPW2 causes recombination defects in humans and mice, including impaired homologous chromosome synapsis and defective DNA double-strand break repair. CUT&Tag analysis revealed that ZCWPW2 exhibits increased enrichment at dual H3K4me3 and H3K36me3 sites in the presence of PRDM9, while binding to promoter regions independently of PRDM9 to regulate meiotic transcription. Mass spectrometry further showed that ZCWPW2 forms a complex with ZCWPW1 and interacts with recombination-associated proteins in a ZCWPW1-dependent manner. Mechanistically, we demonstrate that the ZCWPW1–ZCWPW2 complex enhances the functions of key lactylation regulators LDHA and EP300, thereby promoting lactylation of recombination-associated proteins and stabilizing their abundance. Collectively, we identify ZCWPW2 as a previously unrecognized but essential factor in meiotic recombination, elucidate the molecular mechanism of the PRDM9/ZCWPW1/ZCWPW2 system in regulating recombination, and uncover a critical role for lactylation in meiosis.

## Graphical Abstract

**Graphical Abstract:**
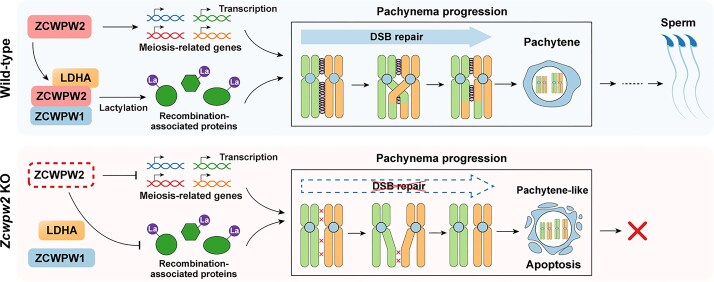

## Introduction

Meiotic recombination plays a key role in ensuring genetic diversity and the precise segregation of homologous chromosomes, both of which are essential for producing viable gametes in reproducing organisms [1]. Disruptions in the recombination process have been strongly implicated in infertility [2]. Meiotic recombination is initiated by SPO11-induced DNA double-strand breaks (DSBs), which are subsequently repaired using the homologous chromosome as a template [3]. This repair either results in reciprocal exchange of flanking regions (crossovers) or proceeds without reciprocal exchange (non-crossovers) at the pachytene stage when the homologous chromosomes become fully synapsed [3]. In most mammals, the DSBs are concentrated in short genomic segments known as “recombination hotspots” [4], the locations of which are determined by PRDM9, a meiosis-specific histone lysine methyltransferase [5]. PRDM9 contains four major domains: an N-terminal KRAB domain involved in regulating protein–protein interactions, an SSXRD domain functioning as a nuclear localization signal, a PR/SET domain responsible for methyltransferase activity, and a C-terminal zinc finger (ZF) array mediating DNA recognition [4–7]. Through its ZF domain that recognizes specific DNA motifs and PR/SET domain that trimethylates H3K4 and H3K36, PRDM9 defines the genomic sites of meiotic recombination initiation [5, 8–12]. Notably, the ZF domain of PRDM9 is highly polymorphic both within and across species, including humans and mice [5, 12–16]. Such polymorphisms alter the DNA-contacting amino acids and/or the number and arrangement of zinc fingers in the DNA-binding domain, thereby modifying PRDM9-binding affinity and leading to substantial differences in hotspot positions and activities within and across species [5, 13, 16–22]. In mice, strains with distinct *Prdm9* alleles (e.g. *Prdm9*^Dom2^, *Prdm9*^Dom3^, and *Prdm9*^Cst^) typically share only 1%–3% of recombination hotspots [23–25], and in humans, hotspot landscapes vary substantially across populations carrying different *PRDM9* variants (e.g. *PRDM9*^A^, *PRDM9*^B^, and *PRDM9*^C^) [12, 15, 18, 19, 26, 27]. It is reported that in *Prdm9* knockout (KO) mice, DSBs occur at promoter regions instead of recombination hotspots, and their inefficient repair disrupts homologous chromosome synapsis, causing meiotic arrest at the zygotene stage [14, 28]. To date, PRDM9 is the only known dual histone methylation writer responsible for directing the specification of hotspots and controlling recombination progression during meiosis in mammals.

After PRDM9 catalyzes the methylation of H3K4 and H3K36 on nucleosomes, these marks are specifically recognized at recombination hotspots by ZCWPW1, a member of the CW domain–containing protein family, through its zf CW and PWWP domains, respectively [29–31]. This recognition was evidenced in cultured HEK293T cells, mouse spermatocytes, and testicular tissues [29–31]. Mice lacking *Zcwpw1* exhibit defective DSB repair and incomplete homologous synapsis [32]. To date, ZCWPW1 remains the only known dual histone methylation reader that facilitates the DSB repair during mammalian meiotic recombination. Nevertheless, the molecular mechanism of the PRDM9-mediated dual histone methylation writer–reader system in orchestrating recombination events remains unclear.

Using a phylogenetic approach, researchers identified candidate genes potentially associated with PRDM9, with *ZCWPW2* showing a strong coevolutionary relationship with *PRDM9* across vertebrates and being predicted to be recruited to PRDM9-binding sites [33]. ZCWPW2 is a paralog of ZCWPW1, containing both zf-CW and PWWP domains [33]. An *in vitro* binding assay revealed that mouse ZCWPW2 binds to both H3K4me3 and H3K36me3, with a stronger binding observed when both marks are present simultaneously [34]. Based on these findings, ZCWPW2 is hypothesized to be a missing link between PRDM9 binding and the recruitment of the recombination machinery.

In this study, we constructed *Zcwpw2* KO mice using CRISPR–Cas9 technology and found that the KO mice were sterile in both sexes, exhibiting synapsis failure and DNA repair defects. Importantly, whole-exome sequencing (WES) identified a loss-of-function mutation in *ZCWPW2* in an infertile man with meiotic arrest. Further CUT&Tag analysis showed that ZCWPW2 is more enriched at H3K4me3- and H3K36me3-marked sites when PRDM9 is present, and forms a complex with ZCWPW1 at these sites. Additionally, ZCWPW2 independently binds to promoter regions, regulating the transcriptional expression of meiosis-related genes, including *ZCWPW1*, as well as the lactylation writer *EP300*. Mass spectrometry profiling demonstrated that the ZCWPW1–ZCWPW2 complex interacts with recombination machinery-associated proteins, such as SYCP1, HSPA2, TEX11, MSH2, MLH1, and MDC1. Moreover, lactate dehydrogenase A (LDHA) was identified as a key interactor of the ZCWPW1–ZCWPW2 complex, and this interaction is critical for sustaining the enzymatic activity of LDHA. Combining lactylome analysis with cellular functional experiments, we further found that the ZCWPW1–ZCWPW2 complex promotes the lactylation of recombination machinery-related proteins, thereby enhancing their stability. In all, our study is the first to demonstrate the crucial role of ZCWPW2 in meiotic recombination in both humans and mice, complementing the histone methylation writer–reader system and elucidating the molecular mechanisms by which this system orchestrates the recombination process, and highlighting a role of protein lactylation in meiotic regulation.

## Materials and methods

### Study participants and genetic analysis

A total of 279 non-obstructive azoospermia (NOA) patients were recruited from West China Second University Hospital. The study was approved by the Ethical Review Board of West China Second University Hospital, Sichuan University, and written informed consent was obtained from all participants in accordance with the Declaration of Helsinki.

Genomic DNA was extracted from peripheral blood using the QIAamp DNA Blood Mini Kit (QIAGEN, 51126). WES was performed using Agilent SureSelect Human All Exon V6 kits on an Illumina HiSeq X-TEN platform. Reads were aligned to the human reference genome (hg38), and variants were filtered and annotated across multiple databases (dbSNP, 1000 Genomes, HGMD, ExAC). Functional prediction tools included PolyPhen-2, SIFT, MutationTaster, CADD, SPIDEX, and DEXSeq. Candidate variants in *ZCWPW2* were confirmed by Sanger sequencing. Primer sequences are listed in Supplementary Table S1.

### Generation of Zcwpw2 KO mice via CRISPR–Cas9 editing

All procedures involving animals followed institutional guidelines and national regulations for laboratory animal welfare. The experimental protocols of animal were approved by the Experimental Animal Management and Ethics Committee of West China Second University Hospital, Sichuan University (approval no. 2021033). The *Zcwpw2* KO mouse model was generated on a C57BL/6 genetic background by CRISPR–Cas9-mediated genome editing. Specifically, exon 3 of the *Zcwpw2* gene was targeted by a single guide RNA (sgRNA; Supplementary Table S2) to introduce a frameshift mutation, resulting in a functional knockout. Mouse zygotes were coinjected with an RNA mixture containing Cas9 mRNA (∼50 ng/μl) and sgRNA (∼30 ng/μl) and transferred to pseudopregnant recipients to generate the F0 generation. Mice were housed under specific pathogen-free (SPF) conditions in a controlled environment (22 ± 2 °C; 40%–60% humidity) with a 12-h light/dark cycle and were provided access to standard chow and water.

Genomic DNA was extracted from tail biopsies of 7-day-old mice, and genotypes were determined by polymerase chain reaction (PCR) amplification followed by Sanger sequencing. The primers for genotypes were provided in Supplementary Table S2.

### Fertility test

Fertility was evaluated by continuous natural mating over 6 months. Sexually mature (8 weeks) *Zcwpw2* KO males and females, and littermate WT controls were paired with WT partners. Vaginal plugs were checked each morning to confirm successful mating. Plug-positive females were separated into individual cages, and the number of pups per litter was recorded to assess fecundity.

### qPCR

Total RNA was extracted from mouse tissues, cultured cells, or human testis samples (obtained with informed consent) using the RNA Easy Fast Tissue/Cell Kit (Tiangen, 4992732), and ∼0.5 μg of RNA was reverse-transcribed into complementary DNA (cDNA) with the PrimeScript RT Reagent Kit (TaKaRa, RR037A). Diluted cDNA (1:10) was used for qPCR with iTaq Universal SYBR Green Supermix (Bio-Rad, 1725124). *Tubulin* was used as an internal control, and relative mRNA levels were calculated using the 2^−ΔΔCt method. Primer sequences are listed in Supplementary Table S1.

### Western blotting and co-immunoprecipitation (Co-IP) assay

Proteins were extracted from mouse and human testes or cultured cells using RIPA lysis buffer (Applygen, C1053) on ice, followed by centrifugation at 14 000 × *g* for 15 min. Equal amounts of protein were separated by 10% sodium dodecyl sulfate–polyacrylamide gel electrophoresis (SDS–PAGE) and transferred onto polyvinylidene fluoride membranes (Millipore, ISEQ00010). Membranes were blocked for 30 min at room temperature and subsequently incubated with primary antibodies overnight at 4°C, followed by secondary antibodies incubation for 1 h at room temperature. All antibodies were diluted in 5% milk in 1× TBST. Signals were detected using the Super ECL Plus Western Blotting Substrate (Applygen, P1050) and imaged with a ChemiDoc MP Imaging System (Bio-Rad, 1708280).

For the Co-IP assay, protein lysates were incubated with primary antibodies overnight at 4°C, followed by incubation with 50 μl Protein A/G Magnetic Beads (Selleck) for 2 h at room temperature. Beads were washed three times with buffer (50 mM Tris–HCl, pH 7.4; 0.1% Triton X-100; 500 mM NaCl), and bound proteins were eluted with 1× SDS loading buffer and boiled at 95°C for 5 min. Samples were then analyzed by SDS–PAGE and immunoblotting. Antibody details are listed in Supplementary Table S3.

### Hematoxylin and eosin (H&E) staining

Human and mouse testes and mouse ovaries were fixed in 4% paraformaldehyde (PFA) overnight at 4 °C. After fixation, samples were rinsed in phosphate-buffered saline (PBS), dehydrated through a graded ethanol series, cleared in xylene, and embedded in paraffin. Paraffin blocks were trimmed and sectioned at a thickness of 5 μm. The sections were baked at 60°C for 1 h, dewaxed in xylene, and rehydrated through descending ethanol gradients to distilled water.

Slides were immersed in hematoxylin solution (Beyotime, C0105M) for nuclear staining, rinsed in running water, differentiated, and blued. This was followed by eosin counterstaining to visualize cytoplasmic structures. After staining, slides were dehydrated through ascending ethanol concentrations, cleared in xylene, and coverslipped with a mounting medium. Sections were imaged using an automated digital slide scanner (Pannoramic MIDI, 3DHISTECH, Hungary).

### Chromosome spreading and immunofluorescence staining

Spermatocyte and oocyte chromosome spreads were prepared as previously described [35]. Seminiferous tubules or embryonic ovaries (E16.5–18.5) were incubated in hypotonic buffer (30 mM Tris, 50 mM sucrose, 17 mM sodium citrate, 5 mM EDTA, 2.5 mM dithiothreitol (DTT), and 1 mM phenylmethylsulfonyl fluoride (PMSF)) for 30 min, transferred to 100 mM sucrose, dissociated, and spread on slides and fixed in 1% PFA with 0.1% Triton X-100.

Slides were blocked for 30 min in antibody dilution buffer (2% normal donkey serum, 0.3% BSA, and 0.01% Triton-X-100 in 1× TBS), incubated with primary antibodies overnight at 37°C, washed, and blocked again for ≥6 h at 4 °C. After incubation with secondary antibodies for 1.5 h at 37°C and final washes, samples were mounted with antifade medium and imaged using a confocal microscope (FV3000, Olympus, Japan). Antibodies are listed in Supplementary Table S3.

### Criteria for synaptic defects

Based on previous studies, synaptic defects mainly include non-homologous chromosome synapsis as well as homologous chromosome asynapsis and desynapsis [36–38]. Non-homologous chromosome synapsis refers to chromosomes that incorrectly paired with non-homologous partners, appearing as branched SYCP3-positive structures rather than normal side-by-side alignment of lateral elements. Asynapsis is defined in pachytene spermatocytes as cases in which homologous chromosomes fail to complete synaptonemal complex. Desynapsis is mainly observed during mid-to-late pachytene, when spermatocytes have already established a synaptonemal complex but prematurely released homologous chromosome pairing before reaching diplotene.

To assess and quantify these defects, SYCP3 was used to label the lateral elements and SYCP1 to mark the central elements of the synaptonemal complex. We quantified WT pachytene spermatocytes and *Zcwpw2* KO pachytene-like spermatocytes by immunofluorescence staining. Spermatocytes showing branched SYCP3 structures were scored as non-homologous chromosome synapsis. Spermatocytes in which homologous chromosomes failed to show SYCP1 signals colocalized with SYCP3 signals were scored as asynapsis.

### Cell culture and transfection

HEK293T cells (CRL-11268; ATCC) and GC-2 cells (CRL-2196; ATCC) were cultured in Dulbecco’s Modified Eagle Medium (DMEM; Gibco, 11965092) supplemented with 10% fetal bovine serum (FBS; Gibco, 12483020). HEK293T and GC-2 cells were treated either with 20 mM sodium oxamate (Santa Cruz, sc-215880), a specific LDHA inhibitor [39], or with 10 μM A-485 (MedChemEpress, HY-107455), an EP300 inhibitor, for 16 h [40]. To activate EP300 activity, cells were treated with 5 μM CTPB (MedChemEpress, HY-124960) for 24 h [41]. Dimethyl sulfoxide (DMSO)-treated cells served as controls in both conditions. Cells were treated with 20 mM lactate (MedChemEpress, HY-B2227) for 14 h.

Full-length cDNAs of *ZCWPW1, ZCWPW2, PRDM9^B^, SYCP1, HSPA2, SYCE1, SPATA22, TEX11, MLH1, MSH2, LDHA*, or *MDC1* were synthesized and cloned into pcDNA™3.1(+) vectors with Flag, HA, or Myc tags by the commercial biotechnology companies PAIVIBIO (China) and TSINGKE (China). CW domain (*ZCWPW2*-Δ*CW* plasmid and *ZCWPW1*-Δ*CW* plasmid), PWWP domain (*ZCWPW2*-Δ*PWWP* plasmid and *ZCWPW1*-Δ*PWWP* plasmid), and CW + PWWP domain (*ZCWPW2*-Δ*CW *+ *PWWP* plasmid and *ZCWPW1*-Δ*CW *+ *PWWP* plasmid) deletion constructs of Flag-*ZCWPW2* and HA-*ZCWPW1* were generated by recombination-based PCR using the full-length Flag-*ZCWPW2* and HA-*ZCWPW1* plasmids as templates. The lactylation site mutant expression plasmids were generated by employing the Fast Mutagenesis System (TransGen Biotech, FM111-01). Primer sequences used for the amplification of mutated plasmids are listed in Supplementary Table S4.

Small interfering RNA (siRNA) constructs targeting *ZCWPW1, Zcwpw1*, and *Zcwpw2* were synthesized by PAIVIBIO (China). Plasmid and siRNA transfection were performed using jetPRIME reagent (Polyplus, 101000046) according to the manufacturer’s instructions. siRNA sequences are provided in Supplementary Table S4.

### Cleavage under targets & tagmentation (CUT&Tag) assay

CUT&Tag was performed as previously described [42]. HEK293T cells transfected with Flag-*ZCWPW2* alone or co-transfected with Flag-*ZCWPW2* and Myc-*PRDM9^B^* were harvested, counted, and centrifuged at 300 × *g* for 5 min at room temperature. Approximately 600 000 cells were incubated with 10 μl Concanavalin A-coated magnetic beads for 15 min at room temperature. The anti-Flag antibody (1:10; Proteintech, 66008-4-Ig) and control IgG were incubated with the bead-cell suspension on a rotator overnight at 4°C. After removal of the primary antibody, cells were incubated with a secondary antibody for 1 h at room temperature. pA-Tn5 transposase was added and incubated for 1 h at room temperature. Tagmentation was performed in the presence of Mg²⁺ at 37°C for 1 h. DNA was purified with 1× AMPure XP beads, washed twice with 80% ethanol, and eluted in 35 μl of 10 mM Tris–HCl (pH 8.0). PCR amplification was performed on 30 μl of eluted DNA. Libraries were sequenced on an Illumina platform by Frasergen Co., Ltd. (Wuhan, China).

Raw FASTQ files were quality-checked and trimmed using FastQC, then aligned to the human reference genome (hg38) with Bowtie2. PCR duplicates were marked and removed using Picard (MarkDuplicates, v2.25.6). Paired-end reads mapped to the same chromosome were filtered using samtools view (V1.12). Peak calling was performed with MACS2 (v3.0.0a6). The PRDM9-binding sites were detected by re-analysis previously published ChIP-seq datasets of HEK293T cell transfected with the human PRDM9 reference allele (the “B” allele). PRDM9-binding sites were defined as peaks detected in the dataset, consistent with the criteria described above.

Purified DNA was quantified by PCR and qPCR to validate Flag-ZCWPW2-binding sites (*RAD51AP1, ZCWPW1, BARD1, RAD51C, MRE11, ATM, ANKRD31, SMC2, RAD21*, and *EP300*). Enrichment was calculated using the Fold Enrichment and Percent Input methods. All assays were performed in triplicate. Primer sequences are listed in Supplementary Table S5.

### Dual-luciferase reporter assays

HEK293T cells were co-transfected with promoter reporter plasmids (pGL3-based constructs promoter regions of *RAD51AP1, ZCWPW1, BARD1, RAD51C, MRE11, ATM, ANKRD31, SMC2, RAD21*, or *EP300*) and the Flag-*ZCWPW2* plasmid. pGL3-control and pGL3-basic vectors were used as positive and negative controls, respectively. After 48 h, luciferase activity was measured using the Dual-luciferase reporter assay (Promega) according to the manufacturer’s protocol. Primer sequences are listed in Supplementary Table S4.

### Immunoprecipitation-mass spectrometry (IP-MS) analysis

Human testis was lysed in RIPA buffer supplemented with 1 mM PMSF and protease inhibitors (Applygen, P1265) on ice. Lysates were incubated with 5 μg anti-ZCWPW2 antibody (Sigma, HPA039839) overnight at 4°C. Detailed Co-IP procedures were described in the Co-IP section above. Immunoprecipitated proteins were separated by SDS–PAGE and visualized by Coomassie Brilliant Blue staining. Gel bands were excised and subjected to in-gel tryptic digestion. Briefly, the gel piece was destained in 50 mM NH_4_HCO_3_/50% acetonitrile (v/v) until fully transparent, then dehydrated in 100% acetonitrile for 5 min and dried. Reduction was performed by rehydrating the gel piece with 10 mM dithiothreitol and incubating at 56°C for 1 h, followed by another dehydration step with 100% acetonitrile. After removal of the solvent, the gel was alkylated with 55 mM iodoacetamide at room temperature in the dark for 45 min, washed with 50 mM NH_4_HCO_3_, and dehydrated again with acetonitrile. The gel piece was then rehydrated on ice with trypsin (10 ng/μl in 50 mM NH_4_HCO_3_) for 1 h, excess solution was removed, and digestion proceeded overnight at 37°C. Peptides were extracted sequentially using 50% acetonitrile/5% formic acid followed by 100% acetonitrile, dried completely, and reconstituted in 2% acetonitrile/0.1% formic acid.

The resulting peptides were then subjected to LC–MS/MS analysis. They were resuspended in mobile phase A and separated using a NanoElute ultrahigh-performance liquid chromatography system (Bruker). Mobile phase A consisted of 0.1% formic acid with 2% acetonitrile in water, and mobile phase B consisted of acetonitrile containing 0.1% formic acid. The chromatographic gradient was programmed as follows: 6%–24% B from 0 to 14 min, 24%–35% B from 14 to 16 min, 35%–80% B from 16 to 18 min, and held at 80% B from 18 to 20 min, at a constant flow rate of 500 nl/min.

Following chromatographic separation, peptides were ionized using a capillary ion source at a spray voltage of 1.75 kV and analyzed on a timsTOF Pro mass spectrometer (Bruker Daltonics) equipped with a trapped ion mobility device. Both precursor ions and their fragment ions were detected in the TOF analyzer. Data acquisition was performed in data-independent acquisition mode using the dia-PASEF workflow. Raw data were searched against the Homo sapiens reference proteome (UniProtKB) using MaxQuant (v1.6.15.0) with its integrated Andromeda search engine under default settings. Label-free quantification (LFQ) values were generated, and the results were processed in two steps: (i) removal of contaminants, reverse hits, and proteins identified by site only, and (ii) normalization of LFQ intensities using the median of commonly identified proteins. Missing values were imputed with the minimum detected intensity.

### Mass spectrometry targeting lactylation profiling

Fresh testes from juvenile *Zcwpw2* KO and WT mice were collected and processed by PTM Bio (Hangzhou, China). Samples were washed with 1× PBS, pulverized using a Cryo-Prep^™^ CP02 system, and snap-frozen in liquid nitrogen. Proteins were extracted by sonication in lysis buffer (8 M urea, 1% protease inhibitor cocktail, 3 μM TSA, and 50 mM NAM), and concentrations were measured using a BCA assay (Beyotime, China). Equal amounts of protein were used for lactylation-modified peptide analyses.

Protein samples were adjusted to equal volumes with lysis buffer. The sample was slowly added to the final concentration of 20% (m/v) TCA to precipitate protein, then vortexed to mix and incubated for 2 h at 4°C. The precipitate was collected by centrifugation at 4500 × *g* for 5 min at 4°C. The precipitated protein was washed with pre-cooled acetone for twice and dried for 1 min. The protein sample was then redissolved in 200 mM TEAB and ultrasonically dispersed. Trypsin was added at 1:50 trypsin-to-protein mass ratio for the first digestion overnight at 37°C The sample was reduced with 5 mM dithiothreitol for 60 min at 37°C and alkylated with 11 mM iodoacetamide for 45 min at room temperature in darkness. Trypsin was added at 1:100 trypsin-to-protein mass ratio for a second 4 h-digestion at 37°C. Finally, the peptides were desalted by Strata X SPE column.

For lactylation profiling, the resulting peptides were dissolved in IP buffer (100 mM NaCl, 1 mM EDTA, 50 mM Tris–HCl, 0.5% NP-40, pH 8.0). The supernatant was transferred to pre-washed anti-L-lactylation antibody beads (PTM-1404, PTM Bio). Samples were incubated with gentle rotation at 4°C overnight to enrich lactylated peptides. Then, the beads were washed for four times with IP buffer and twice with H_2_O. Peptides were eluted, vacuum-dried, desalted using C18 ZipTips (Millipore), and subjected to LC–MS/MS. Peptides were loaded onto Evotips and separated on an Evosep One system using the Whisper 40SPD method with mobile phases consisting of 0.1% formic acid in water (A) and in acetonitrile (B). Separated peptides were ionized at 1.5 kV and analyzed on a timsTOF Pro2 mass spectrometer using dia-PASEF acquisition. Significant differences were defined as *P* < 0.05 with a fold change ≥ 1.2, based on the lactylation abundance of lactylation sites between groups.

For proteins which expression levels and lactylation signals were both reduced, lactylation intensities and the corresponding immunoprecipitated protein signals were quantified from western blotting images using ImageJ [43, 44]. Lactylation efficiency was calculated as the ratio of lactylation signal to immunoprecipitated protein signal, and a KO/WT ratio was obtained:


\begin{eqnarray*}
&&{\mathrm{Ratio}}=\\&& \frac{{KO\left( {\textit{lactylation}\ \textit{signal}/{\mathrm{immunoprecipitated\ protein\ signal}}} \right)}}{{WT\left( {\textit{lactylation}\ \textit{signal}/{\mathrm{immunoprecipitated\ protein\ signal}}} \right)}}\\&=& \frac{{KO\left( {\textit{lactylation}\ \textit{efficiency}} \right)}}{{WT\left( {\textit{lactylation}\ \textit{efficiency}} \right)}}
\end{eqnarray*}

Values of ratio < 1 indicate that the observed downregulation of lactylation is not due to reduced protein abundance but rather reflects a selective decrease in the lactylation efficiency.

### L-LDH activity assay

L-LDH activity was measured using the L-LDH assay kit (Beyotime, P0393), according to the manufacturer’s protocol. Briefly, cells or testicular tissues were homogenized in Buffer A on ice and centrifuged at 12 000 × *g* for 5 min at 4°C. Cell or testis supernatants were diluted 50- to 100-fold. For each reaction, 50 μl of sample or standard (0.3–50 mU/ml) was mixed with 50 μl of freshly prepared WST-8 working solution. Reactions were incubated at 37°C for 30 min, and absorbance was measured at 450 nm using a microplate reader (Thermo Fisher, USA).

### Quantification of lactate levels

Cell or tissue lysates were extracted from equal amounts of cultured cells and testis tissue using metabolic assay lysis buffer (Beyotime, S0208S). Lactate concentrations in both tissue/cell lysates were measured using a Biochemical and Immunoassay Analyzer (Atellica, Siemens) at the Department of Laboratory Medicine, West China Second University Hospital.

### Statistical analysis

Data are presented as mean ± SEM. Statistical analyses were performed using SPSS 20.0. The number of independent biological replicates is indicated in the figure legends. Comparisons between two groups were conducted using a two-tailed Student’s *t*-test. Statistical significance was defined as *P < 0.05.

## Results

### Spatiotemporal expression and localization of PRDM9, ZCWPW1, and ZCWPW2 during mammalian germ cell development

**Figure 1.**
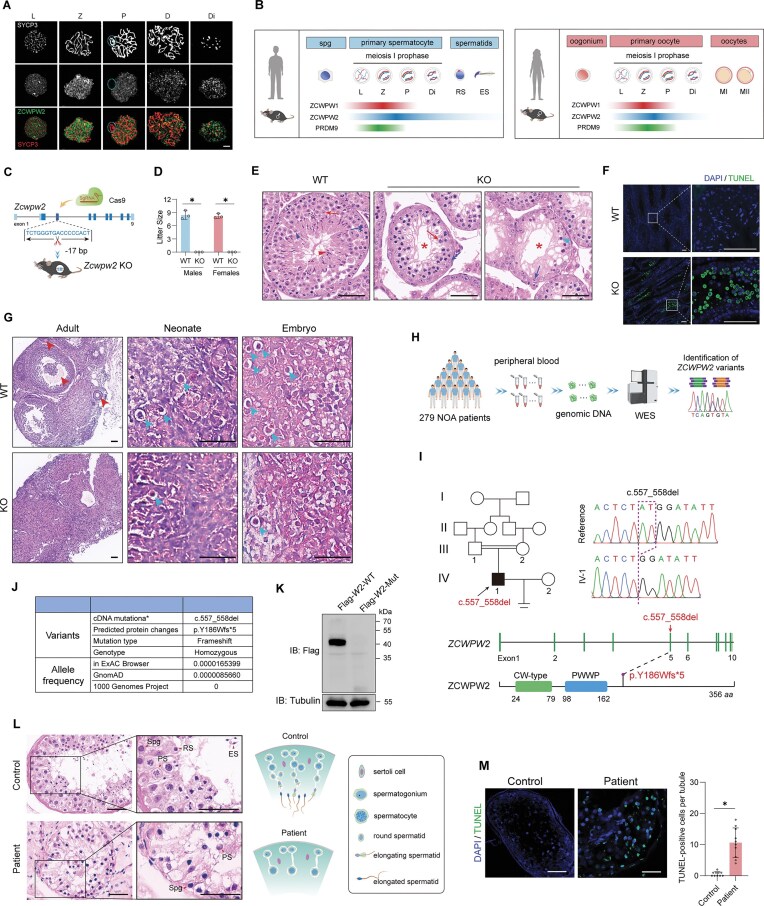
ZCWPW2 is indispensable for meiotic progression in both humans and mice. (**A**) Immunofluorescence of chromosome spreads showing ZCWPW2 (green) and SYCP3 (red) in WT spermatocytes at different meiotic stages. L, Leptotene; Z, Zygotene; P, Pachytene; D, Diplotene; Di, Diakinesis; scale bar: 5 μm. (**B**) Schematic overview of PRDM9/ZCWPW1/ZCWPW2 expression during germ cell development in human and mouse testes and ovaries. Spg, spermatogonia; L, Leptotene; Z, Zygotene; P, Pachytene; D, Diplotene; Di, Diakinesis; RS, round spermatids; ES, elongating/elongated spermatids; MI, meiosis metaphase I; MII, meiosis metaphase II. (**C**) Schematic representation of a 17-bp deletion in exon 3 of *Zcwpw2* generated via CRISPR–Cas9 genome editing to produce knockout mice. (**D**) Litter size was assessed by natural mating over a 6-month period (*n* = 3 biologically independent adult WT mice and KO mice; Two-tailed Student’s *t*-test; *P < 0.05; error bars, s.e.m.). (**E**) H&E staining of testis sections from adult WT and *Zcwpw2* KO mice. Blue arrow, spermatogonia; Red arrow, spermatocytes; Blue arrowhead, round spermatids; Red arrowhead, elongating spermatids; Cyan arrowhead, apoptotic cells; Asterisk, lumen of the seminiferous tubule; scale bar: 50 μm. (**F**) TUNEL staining (green) and DAPI counterstaining (blue) of testis sections from adult WT and *Zcwpw2* KO mice; scale bar: 50 μm. (**G**) H&E staining of ovarian sections from WT and *Zcwpw2* KO females at the embryonic (E15.5), neonatal (P0), and adult (8-week) stages. Red arrowhead, follicles at different developmental stages. Cyan arrowhead, primordial follicles; scale bar: 50 μm. (**H**) Schematic illustrating the process of screening *ZCWPW2* variants in infertile men with NOA using WES. (**I**) Pedigree analysis and Sanger sequencing of the patient carrying a homozygous *ZCWPW2* frameshift variant (c.557_558del). (**J**) Summary of the *ZCWPW2* frameshift variant (c.557_558del) detected in the NOA patient and its rarity in population databases (*The NCBI reference sequence for *ZCWPW2* was NM_001324169.2.). (**K**) Western blotting analysis of HEK293T cells transfected with WT or mutant Flag-*ZCWPW2* (c.557_558del). (**L**) H&E staining of testis sections from control and the patient carrying the *ZCWPW2* mutation; scale bar: 50 μm. Spg, spermatogonia; PS, spermatocytes; RS, round spermatids; ES, elongating/elongated spermatids. (**M**) TUNEL staining (green) with DAPI counterstaining (blue) of testicular sections from control and the patient; scale bar: 50 μm (two-tailed Student’s *t*-test; *P < 0.05; error bars, s.e.m.).

In adult mice, we found that *Zcwpw2* was highly expressed in testes (Supplementary Fig. S1A), with a pattern similar to *Prdm9* [45] yet distinct from the broad expression of *Zcwpw1* [32]. In fetal ovaries, *Zcwpw2* reached its maximum at embryonic day 15.5 (E15.5), while *Zcwpw1* peaked at E14.5; however, both were nearly undetectable during E11.5 to E13.5 and E16.5 to E17.5 (Supplementary Fig. S1B). In fetal testes, *Zcwpw2* and *Zcwpw1* were minimally expressed, but both rose markedly from postnatal day 10 (P10) (Supplementary Fig. S1C). *Zcwpw2* peaked at P20 and stabilized around P25, whereas *Zcwpw1* peaked earlier at P15 and subsequently stabilized at a low level (Supplementary Fig. S1C). Their expression patterns differ slightly from that of *Prdm9*, which is transiently upregulated in fetal ovaries from E13.5 to E16.5 but remains nearly undetectable in fetal testes, while its expression in neonatal testes progressively increases between P10 and P18 [45]. Chromosome spread experiments in spermatocytes further revealed that ZCWPW2 and ZCWPW1 exhibited similar expression during the leptotene and zygotene stages (Fig. 1A and Supplementary Fig. S1D), resembling the pattern of PRDM9 [46]. However, ZCWPW1 localized to the sex body during pachytene and diplotene and disappeared at diakinesis (Supplementary Fig. S1D), whereas ZCWPW2 remained excluded from the sex body and persisted through diakinesis (Fig. 1A). These findings suggested that all three genes exhibit high expression during the zygotene stage of meiosis in both testis and ovary.

In humans, these three genes show expression patterns similar to those in mice: in ovaries, they are primarily expressed during the embryonic stage (12–18 weeks post conception, WPC) but show low or nearly undetectable levels in adulthood, whereas in testes they are nearly absent during embryogenesis and highly expressed in adulthood (Supplementary Fig. S1E–L).

Moreover, we enzymatically dissociated testicular tissue from consenting patients with obstructive azoospermia as well as from WT mice, and obtained germ cells at different developmental stages (Supplementary Fig. S2A). Immunofluorescence staining revealed that ZCWPW1 and PRDM9 were exclusively expressed in spermatocytes (Supplementary Fig. S2B and C), while ZCWPW2 was additionally detected in the head and tail regions of spermatids (Supplementary Fig. S2D). The localization patterns of all three proteins in humans were consistent with those observed in different germ cell stages of mice by immunofluorescence staining analysis (Supplementary Fig. S2E) [47, 48]. Together, these results demonstrated that PRDM9, ZCWPW1, and ZCWPW2 share highly similar expression patterns, with all three being prominently expressed during the zygotene stage of meiosis, while ZCWPW2 remains detectable in spermatids (Fig. 1B).

### ZCWPW2 is required for meiotic progression in humans and mice

In this study, we employed CRISPR–Cas9 technology to generate *Zcwpw2* KO mice on the C57BL/6 (B6) background, which carry the *Prdm9*^Dom2^ allele (Fig. 1C and Supplementary Fig. S3A–C). The KO mice were viable and appeared to develop normally, but were infertile in both sexes (Fig. 1D). The KO males displayed significantly reduced testis and epididymis sizes compared to WT controls (Supplementary Fig. S3D and E), with histological analysis and immunofluorescence staining revealing spermatocyte arrest accompanied by apoptotic cells (Fig. 1E and F; Supplementary Fig. S3F–H). In adult females, WT ovaries displayed normal follicular development, whereas no growing follicles were observed in the KO ovaries (Fig. 1G). In WT embryonic ovaries (E15.5), germ cells were primarily organized as germ cell cysts and primordial follicles, while KO embryonic ovaries exhibited a marked reduction in primordial follicles (Fig. 1G). Moreover, in WT neonatal ovaries, germ cells were almost exclusively present as primordial follicles, but their numbers were markedly reduced in KO neonatal ovaries (Fig. 1G). These results indicated that gametogenesis was disrupted at the meiotic stage in both male and female *Zcwpw2* KO mice.

Notably, through WES, we screened for *ZCWPW2* variations in 279 primary NOA patients, and identified a homozygous frameshift variation (c.557_558del) in one patient from a consanguineous family (Fig. 1H and I, and Supplementary Table S6). This *ZCWPW2* variation was either absent or present at extremely low frequencies in most human populations, according to the ExAC Browser, gnomAD, and 1000 Genomes Project databases (Fig. 1J). Sanger sequencing further confirmed that this variation was inherited from the patient’s heterozygous parents (Fig. 1I). Western blotting results showed that this variation led to a lack of ZCWPW2 abundance in transfected cells with mutant plasmids (Fig. 1K). Moreover, histopathological analysis of this patient’s testicular biopsy found that the seminiferous tubules contained only spermatogonia and spermatocytes, with absence of spermatids and evident apoptotic cells (Fig. 1L and M). Therefore, these findings suggested that the absence of ZCWPW2 leads to meiotic arrest in humans and mice.

### Zcwpw2 KO mice exhibit more severe impairment of meiotic recombination than Zcwpw1 KO mice

**Figure 2.**
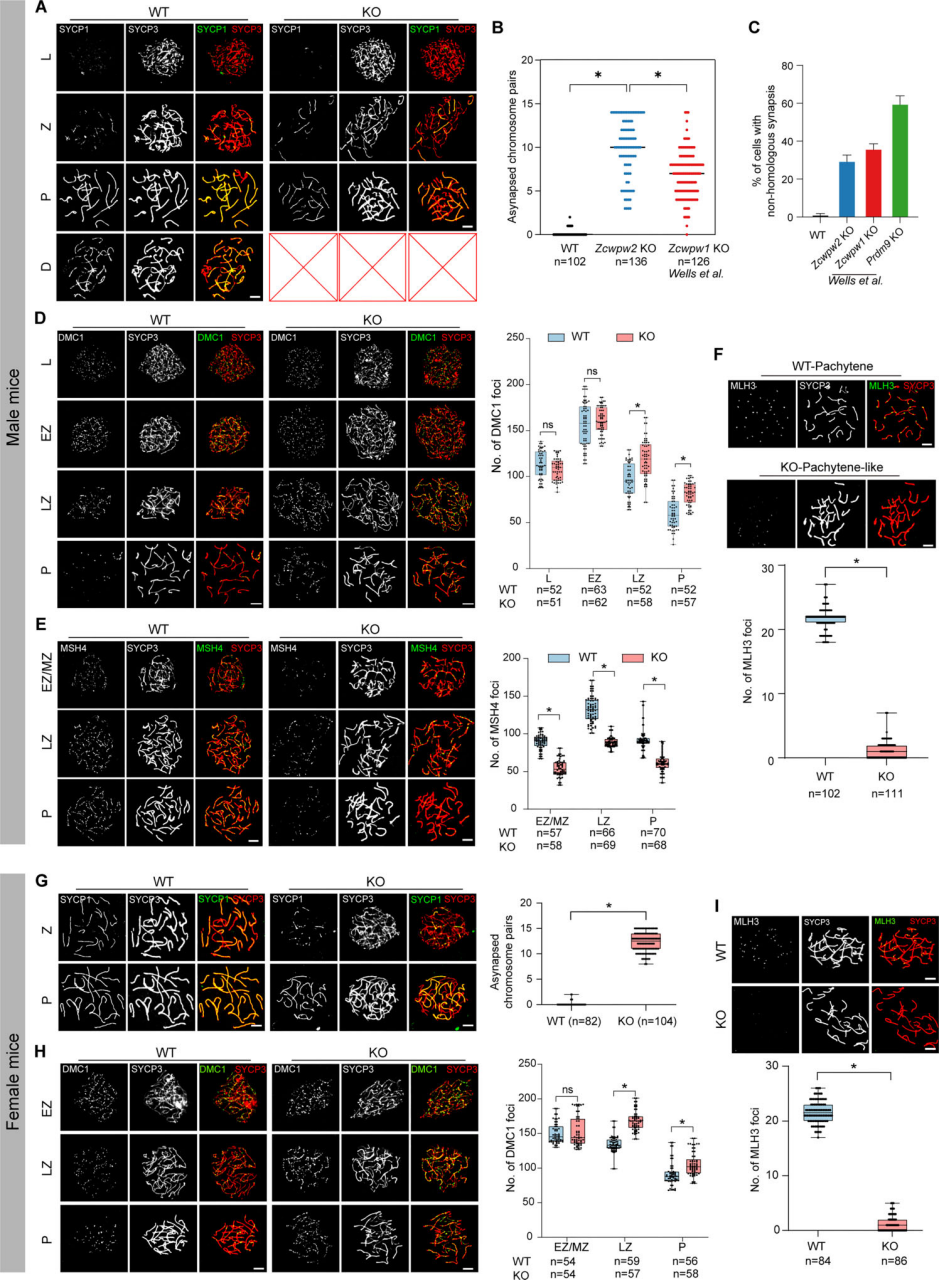
Meiotic recombination defects in *Zcwpw2*-deficient spermatocytes and oocytes. (**A**) Immunofluorescence staining of chromosome spreads from WT and *Zcwpw2* KO spermatocytes at different meiotic stages using SYCP1 (green) and SYCP3 (red) as synaptonemal complex markers. L, Leptotene; Z, Zygotene; P, Pachytene or Pachytene-like; D, Diplotene; scale bar: 5 μm (*n* = 3 biologically independent WT mice and KO mice). (**B**) Dot plot showing the number of asynapsed chromosome pairs per pachytene or pachytene-like spermatocyte in WT, *Zcwpw2* KO, and *Zcwpw1* KO testes (Well *et al.*, 2020); *n*, the number of spermatocytes (two-tailed Student’s *t*-test; *P < 0.05; error bars, s.e.m.). (**C**) Bar graph showing the percentage of spermatocytes exhibiting non-homologous chromosome synapsis in WT, *Zcwpw2* KO, *Zcwpw1* KO, and *Prdm9* KO mice (Well *et al.*, 2020) (*n* = 3 biologically independent WT mice and KO mice; error bars, s.e.m.). (**D**) Immunofluorescence staining of DMC1 (green) and SYCP3 (red) in chromosome spreads from WT and *Zcwpw2* KO spermatocytes at the leptotene (L), early zygotene (EZ), late zygotene (LZ), and pachytene or pachytene-like (P) stages; scale bar: 5 μm; *n*, the number of spermatocytes (two-tailed Student’s *t*-test; *P < 0.05; ns, no significance; error bars, s.e.m.). (**E**) Immunofluorescence staining of MSH4 (green) and SYCP3 (red) in chromosome spreads from WT and *Zcwpw2* KO spermatocytes at early/mid zygotene (EZ/MZ), late zygotene (LZ), and pachytene or pachytene-like (P) stages; scale bar: 5 μm; *n*, the number of spermatocytes (two-tailed Student’s *t*-test; *P < 0.05; error bars, s.e.m.). (**F**) Immunofluorescence staining of MLH3 (green) and SYCP3 (red) in pachytene stage or pachytene-like stage spermatocytes from WT and *Zcwpw2* KO mice; scale bar: 5 μm; *n*, the number of spermatocytes (two-tailed Student’s *t*-test; *P < 0.05; error bars, s.e.m.). (**G**) Immunofluorescence staining of chromosome spreads from WT and KO oocytes at zygotene (Z) and pachytene or pachytene-like (P) stages using antibodies against SYCP1 (green) and SYCP3 (red); scale bars: 5 μm; *n*, the number of oocytes (two-tailed Student’s *t*-test; *P < 0.05; error bars, s.e.m.). (**H**) Immunofluorescence staining of DMC1 (green) and SYCP3 (red) in WT and KO oocytes at early (EZ), late zygotene (LZ), and pachytene or pachytene-like (P) stages; scale bars: 5 μm; *n*, the number of oocytes (two-tailed Student’s *t*-test; *P < 0.05; ns, no significance; error bars, s.e.m.). (**I**) Immunofluorescence staining of MLH3 (green) and SYCP3 (red) in WT and KO oocytes at the pachytene or pachytene-like stage (top), with accompanying quantification of MLH3 foci (bottom); scale bars: 5 μm; *n*, the number of oocytes (two-tailed Student’s *t*-test; *P < 0.05; error bars, s.e.m.).

According to previous studies, synaptic defects mainly include non-homologous chromosome synapsis as well as homologous chromosome asynapsis and desynapsis [36–38]. Notably, in *Zcwpw2* KO males, most spermatocytes exhibited homologous chromosome asynapsis, while a smaller proportion displayed non-homologous chromosome mispairing at the pachytene-like stage (defined by more than five synapsed homologous chromosome pairs per cell) (Fig. 2A, and Supplementary Fig. S4A and B). Despite these defects, synapsis initiation appeared normal, as evidenced by the proper early emergence of SYCP1 during the zygotene stage (Fig. 2A). We further found that the average number of chromosomes showing asynapsis per spermatocyte in *Zcwpw2* KO testes was higher than that observed in *Zcwpw1* KO testes [29] (Fig. 2B), suggesting a more severe synapsis defect in the absence of ZCWPW2. While *Prdm9* KO mice exhibited pronounced synaptic defects, primarily manifested as non-homologous chromosome synapsis [45], such abnormalities occurred at lower frequencies in both *Zcwpw1* and *Zcwpw2* KO spermatocytes (Fig. 2C).

Consistent with observations in *Zcwpw1* KO and *Prdm9* KO lines [32, 45], the *Zcwpw2* KO spermatocytes exhibited no significant difference in γH2AX distribution at the leptotene stage compared with WT spermatocytes, suggesting that DSB formation was unaffected (Supplementary Fig. S4C). However, at the zygotene and pachytene-like stages, γH2AX staining was aberrantly retained on all chromosomes in *Zcwpw2* KO spermatocytes, whereas in WT spermatocytes, it disappeared from synapsed autosomes and concentrated around the sex chromosomes to form the sex body (Supplementary Fig. S4B). These findings suggested a failure in DSB repair in *Zcwpw2* KO spermatocytes, as evidenced by the increased accumulation of recombination-associated DNA repair proteins DMC1, RAD51, and RPA2 at the zygotene and pachytene-like stages compared with WT spermatocytes (Fig. 2D, and Supplementary Fig. S4D and E). As a result, the signals of recombination intermediates MSH4 and TEX11 were diminished in *Zcwpw2* KO spermatocytes at the zygotene and pachytene-like stages compared with WT spermatocytes (Fig. 2E and Supplementary Fig. S5A). Consistent with expectations, MLH1 and MLH3 foci, which serve as markers for crossover sites, were undetectable in *Zcwpw2* KO spermatocytes (Fig. 2F and Supplementary Fig. S5B). In *Prdm9* KO spermatocytes, DMC1, a recombination site-localizing protein, showed minimal localization within γH2AX-stained regions [45]. However, *Zcwpw2* KO spermatocytes exhibited robust co-localization of DMC1 foci with γH2AX signals (Supplementary Fig. S5C), indicating that ZCWPW2 is dispensable for DSB formation. Consistently, we detected comparable recombination defects in *Zcwpw2* KO females (Fig. 2G–I and Supplementary Fig. S6A–C). Collectively, our findings revealed a vital role of ZCWPW2 in the meiotic process in the involvement of regulating recombination machinery.

### ZCWPW2 exhibits increased enrichment at H3K4me3- and H3K36me3-marked sites in the presence of PRDM9 and is found in complex with ZCWPW1 at these sites

**Figure 3.**
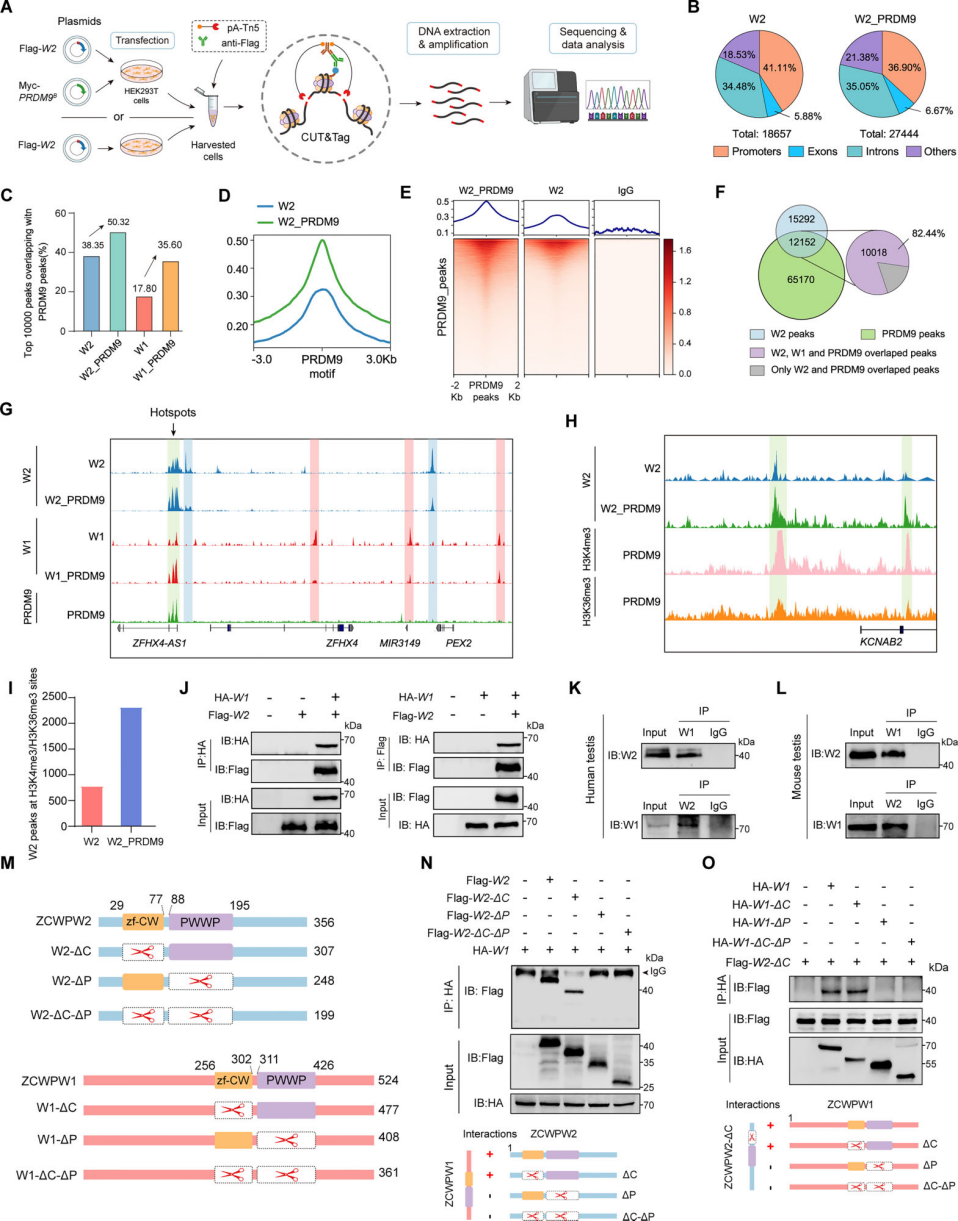
ZCWPW2 shows enhanced enrichment at sites co-marked by H3K4me3 and H3K36me3 in the presence of PRDM9 and is found in complex with ZCWPW1 at these sites. (**A**) Schematic overview of CUT&Tag profiling of ZCWPW2 genomic binding sites in HEK293T cells transfected with Flag-*ZCWPW2 (W2)* alone or co-transfected with Myc-*PRDM9^B^*. (**B**) Pie charts showing the genomic annotation of ZCWPW2 (W2) binding peaks in HEK293T cells transfected with Flag-*ZCWPW2* alone (left) or co-transfected with Myc-*PRDM9^B^* (right). (**C**) Bar graph showing the percentage of the top 10 000 ZCWPW2 (W2) or ZCWPW1 (W1) peaks overlapping with PRDM9-binding sites in the presence or absence of PRDM9. (**D**) Line plot showing ZCWPW2 (W2) binding signal centered around PRDM9 binding motifs (±3 kb) in HEK293T cells expressing Flag-ZCWPW2 with or without Myc-PRDM9. (**E**) Heatmaps and average signal plots showing ZCWPW2 (W2) enrichment centered on PRDM9-binding peaks (±2 kb) in HEK293T cells transfected with Flag-*ZCWPW2* alone, co-transfected with Myc-*PRDM9^B^*, or IgG control. (**F**) Venn diagram showing the overlap among ZCWPW2 (W2), ZCWPW1 (W1), and PRDM9 peaks in HEK293T cells co-expressing Myc-PRDM9. (**G**) Genome tracks snapshot showing representative genomic regions with binding profiles of ZCWPW2 (W2), ZCWPW1 (W1), and PRDM9 in HEK293T cells. (**H**) Genome track snapshots showing ZCWPW2 (W2) signals (green) in HEK293T cells co-expressing Flag-ZCWPW2 and Myc-PRDM9, and ZCWPW2 (W2) signals (blue) in cells expressing Flag-ZCWPW2 alone, together with tracks for PRDM9-induced H3K4me3 (pink) and H3K36me3 (orange). (**I**) ZCWPW2 peaks at H3K4me3/H3K36me3 sites in HEK293T cells co-expressing Flag-ZCWPW2 and Myc-PRDM9, as well as in cells expressing Flag-ZCWPW2 alone. (**J**) Co-IP assays were performed in HEK293T cells co-transfected with HA-*ZCWPW1* (W1) and Flag-*ZCWPW2* (W2). (K and L) Co-IP assays were performed on human (**K**) and mouse (**L**) testis lysates to detect endogenous interactions between ZCWPW1 (W1) and ZCWPW2 (W2). (**M**) Schematic overview showing full-length *ZCWPW2* (*W2*) and ZCWPW1(*W1*), along with their domain-deletion variants lacking either the zf-CW domain (ΔC), the PWWP domain (ΔP), or both (ΔC-ΔP), were generated and were co-transfected in HEK293T cells. (**N**) Co-IP analysis showing the interaction between full-length ZCWPW1 (W1) and full-length or domain-deletion variants of ZCWPW2 (W2). (**O**) Co-IP analysis of ZCWPW2 containing the PWWP domain (W2-ΔC) and full-length or domain-deletion variants of ZCWPW1 (W1) in HEK293T cells. Data for ZCWPW1 ChIP-seq in HEK293T cells, either transfected alone or co-transfected with PRDM9, were obtained from the GEO repository under accession number GSE141516. ChIP-seq data for PRDM9 in HEK293T cells were retrieved from GEO accession GSE99407.

Because no suitable commercial anti-ZCWPW2 antibodies are available for chromatin immunoprecipitation, we conducted CUT&Tag analysis using an anti-Flag antibody in HEK293T cells transfected with full-length human Flag-*ZCWPW2*, either alone or together with human Myc-*PRDM9* (NM_020227.4, corresponding to the *PRDM9*^B^ variant) (Fig. 3A), to characterize ZCWPW2-binding sites in the genome, following previous studies [29, 49]. The antibody binding was specific compared to the IgG control (Supplementary Fig. S7A). A total of 18 657 peaks were identified in cells expressing ZCWPW2 alone, with enrichment in proximal promoters (from –3000 bp to +3000 bp of transcriptional start sites; 41.11%), exons (5.88%), and introns (34.48%) (Fig. 3B). Notably, upon co-transfection with *PRDM9*, the number of ZCWPW2 peaks increased to 27 444 peaks, with 36.90% peaks at promoters, 6.67% peaks in exons, and 35.05% peaks in introns (Fig. 3B).

We first examined the relationship between ZCWPW2-binding sites identified in our study and PRDM9-binding sites reanalyzed from previously published data [49]. In the absence of PRDM9, numerous ZCWPW2 peaks were distributed throughout the genome, including at PRDM9 sites (Fig. 3C). In contrast, co-transfection with *PRDM9* resulted in increased ZCWPW2 occupancy at PRDM9-binding sites (Fig. 3C). Moreover, ZCWPW2 was significantly enriched at PRDM9 sites in the presence of PRDM9, with stronger enrichment at sites bound more abundantly by PRDM9, compared to cells lacking PRDM9 (Fig. 3D and E, and Supplementary Fig. S7B). In addition, we reanalyzed the published data [29] and observed a similar result in which ZCWPW1 peaks overlapped with more PRDM9 sites in the presence of PRDM9 than in its absence (Fig. 3C). We further found that, in the presence of PRDM9, the vast majority of ZCWPW2 peaks at PRDM9 sites overlapped with ZCWPW1 peaks at the same sites (Fig. 3F and G). Reciprocally, in the absence of PRDM9, their peaks diverged and showed lower enrichment compared to those associated with PRDM9 binding (Fig. 3G). These findings suggested that ZCWPW2 binding can be significantly reprogrammed by PRDM9, similar to ZCWPW1.

As expected, in the presence of PRDM9, ZCWPW2 peaks showed a marked increase in enrichment at H3K4me3 sites compared with the absence of PRDM9 (Fig. 3H). Similarly, the presence of PRDM9 resulted in a substantial enhancement of ZCWPW2 binding at H3K36me3 sites compared with its absence (Fig. 3H). In addition, ZCWPW2 binding was found to correlate positively with the levels of both H3K4me3 and H3K36me3 marks in the presence of PRDM9 (Fig. 3H and Supplementary Fig. S7C–E). Importantly, in the presence of PRDM9, more ZCWPW2 peaks overlapped with both H3K4me3 and H3K36me3 sites compared with the absence of PRDM9, showing nearly a twofold increase (Fig. 3I). Therefore, PRDM9 can significantly increase the number of ZCWPW2 peaks that overlap with both H3K4me3 and H3K36me3 sites.

Given that both ZCWPW1 and ZCWPW2 show increased enrichment at sites marked by both H3K4me3 and H3K36me3 in the presence of PRDM9, it is speculated that they may form a complex. The Co-IP assay further confirmed their interaction in HEK293T cells transfected with HA-*ZCWPW1* and Flag-*ZCWPW2* plasmids (Fig. 3J). Their binding was further observed in both human and mouse testis tissues (Fig. 3K and L). A series of truncated variants of ZCWPW2 and ZCWPW1, lacking either the zf-CW domain, the PWWP domain, or both, were constructed with epitope tags and co-transfected into HEK293T cells (Fig. 3M). Co-IP assays revealed that the PWWP domain is critical for the interaction between ZCWPW1 and ZCWPW2, and that deletion of this domain in either protein completely abolished their binding (Fig. 3N and O). Therefore, ZCWPW1 and ZCWPW2 may form a complex through the PWWP domain at dual H3K4me3 and H3K36me3 sites.

### ZCWPW2 binds independently to promoter regions related to meiotic-associated gene transcription

**Figure 4.**
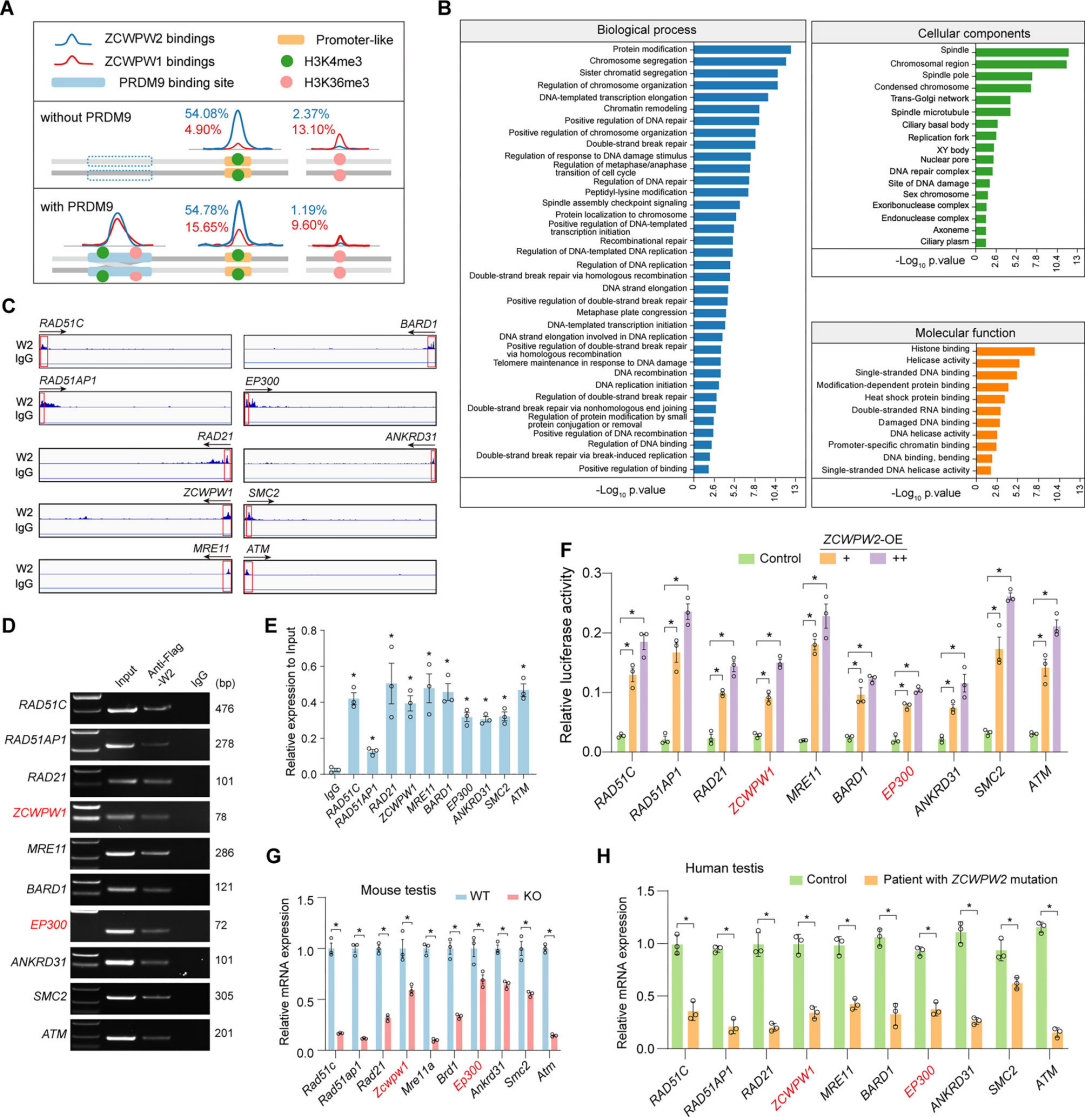
ZCWPW2 binds to H3K4me3-mediated promoter regions in independence of PRDM9. (**A**) Schematic summary illustrating that ZCWPW2 binds strongly to H3K4me3-marked promoter regions both in the absence and presence of PRDM9. (**B**) GO analysis of genes marked by ZCWPW2 peaks at promoter-associated H3K4me3 sites in HEK293T cells. (**C**) CUT&Tag tracks display ZCWPW2 (W2) binding peaks at promoter regions of genes implicated in DNA repair (e.g.* RAD51AP1, RAD51C, MRE11, ATM, BARD1, ZCWPW1*) and chromosome segregation (e.g.* SMC2, RAD21, ANKRD31*), as well as lactylation modification (e.g.* EP300*). IgG was used as a negative control. (**D**) Confirmation of ZCWPW2 (W2) binding at key genes’ promoter regions by PCR amplification using primers targeting the promoter regions. IgG served as a negative control. (**E**) Relative enrichment of ZCWPW2 binding chromatin fragments was measured by qPCR using primers targeting the promoter regions (*n* = 3 independent experiments; Two-tailed Student’s *t*-test; *P < 0.05; error bars, s.e.m.). (**F**) Relative luciferase activity driven by promoter fragments of *RAD51C, RAD51AP1, RAD21, ZCWPW1, MRE11, BARD1, EP300, ANKRD31, SMC2*, and *ATM* in HEK293T cells co-transfected with increasing amounts of Flag-*ZCWPW2* (ZCWPW2-OE, + and ++), compared to the control (empty vector) (*n* = 3 independent experiments; two-tailed Student’s *t*-test; *P < 0.05; error bars, s.e.m.). (**G**) qPCR analysis showing significant downregulation of *Rad51c1, Rad51ap1, Rad21, Zcwpw1, Mre11a, Brd1, Ep300, Ankrd31, Smc2*, and *Atm* mRNA in testes from adult *Zcwpw2* KO mice compared to WT mice (*n* = 3 biologically independent WT mice and KO mice; Two-tailed Student’s *t*-test; *P < 0.05; error bars, s.e.m.). (**H**) Bar graphs showing relative mRNA expression levels of key meiosis-related genes in testicular biopsies from the *ZCWPW2* mutant patient compared to controls (*n* = 3 independent experiments; two-tailed Student’s *t*-test; *P < 0.05; error bars, s.e.m.).

Intriguingly, in the absence of PRDM9, 54.08% of ZCWPW2 peaks were enriched at H3K4me3-binding sites, with 92.93% of these peaks located in the promoter regions (Fig. 4A). In the presence of PRDM9, even after excluding ZCWPW2 enrichment at H3K4me3 sites located within dual histone methylation sites, the remaining 54.78% of ZCWPW2 peaks still showed substantial enrichment at isolated H3K4me3-binding sites, of which 74.69% were located within promoter regions (Fig. 4A). Moreover, more than half of the genes associated with promoter-bound ZCWPW2 peaks were commonly identified under both PRDM9-positive and PRDM9-negative conditions. Concurrently, our above findings showed that ZCWPW2 did not localize to the sex body (Fig. 1A), where sex chromosomes are transcriptionally inactive except for the Pseudoautosomal region, and H3K4me3, typically enriched in transcription activation regions, is also not detected in the sex body [50]. By contrast, regardless of the presence of PRDM9, the enrichment of ZCWPW1 peaks at isolated H3K4me3 sites was much lower than that of ZCWPW2, with only 4.90% of peaks in the absence of PRDM9 and 15.65% in its presence (Fig. 4A). This observation was consistent with previous findings that ZCWPW1 localized to the sex body, where H3K4me3 is nearly absent [50]. Moreover, irrespective of PRDM9 presence, the enrichment of ZCWPW2 peaks at H3K36me3 sites remained low (2.37% without PRDM9; 1.19% with PRDM9) (Fig. 4A), as did the enrichment of ZCWPW1 peaks (13.10% without PRDM9; 9.60% with PRDM9) (Fig. 4A). Collectively, ZCWPW2 peaks displayed substantial enrichment at the H3K4me3-binding sites, independent of PRDM9 presence.

Strikingly, GO analysis revealed that most genes with ZCWPW2 binding peaks in their promoter regions were predominantly enriched for meiotic processes, particularly those related to DNA repair and recombination (Fig. 4B), such as *RAD51AP1, ZCWPW1, BARD1, RAD51C, MRE11, ATM*, and *ANKRD31*, as well as related to homologous chromosome segregation, including *SMC2* and *RAD21* (Fig. 4C and Supplementary Fig. S8) [30, 51–58]. Of note, ZCWPW2 binding at *ZCWPW1* promoter region suggested that ZCWPW2 may regulate *ZCWPW1* expression at the transcriptional level (Fig. 4C). In addition to the gene enrichment associated with DNA recombination machinery, genes related to protein modifications were particularly enriched. Notably, among the 145 modification-related genes, *EP300—*encoding an acyltransferase recognized as a key lysine lactylation writer—stood out [59], exhibiting pronounced abundance.

Then, purified DNA from the CUT&Tag experiment was analyzed by PCR and qPCR to validate ZCWPW2 binding to the promoter fragments of *RAD51AP1, ZCWPW1, BARD1, RAD51C, MRE11, ATM, ANKRD31, SMC2, RAD21*, and *EP300* (Fig. 4D and E). Furthermore, a dual-luciferase reporter assay revealed that the luciferase activity of constructs containing the promoter fragments of *RAD51AP1, ZCWPW1, BARD1, RAD51C, MRE11, ATM, ANKRD31, SMC2, RAD21*, and *EP300* increased in correlation with ZCWPW2 overexpression in HEK293T cells (Fig. 4F). As expected, the expression levels of these meiosis-related genes and *EP300*/*Ep300* were significantly diminished in the testes of *Zcwpw2* KO mice and the *ZCWPW2* mutant patient compared to those observed in controls (Fig. 4G and H). Given that ZCWPW2 lacks both DNA-binding ability and a transcriptional activation domain, we speculated that it may interact with transcriptional co-factors at promoters to promote gene transcription (as mentioned below). Taken together, these results suggested that the transcriptional regulatory function of ZCWPW2 may account for the relatively more severe meiotic phenotype observed in *Zcwpw2* KO mice compared with *Zcwpw1* KO mice.

### The ZCWPW1–ZCWPW2 complex interacts with recombination-associated proteins

**Figure 5.**
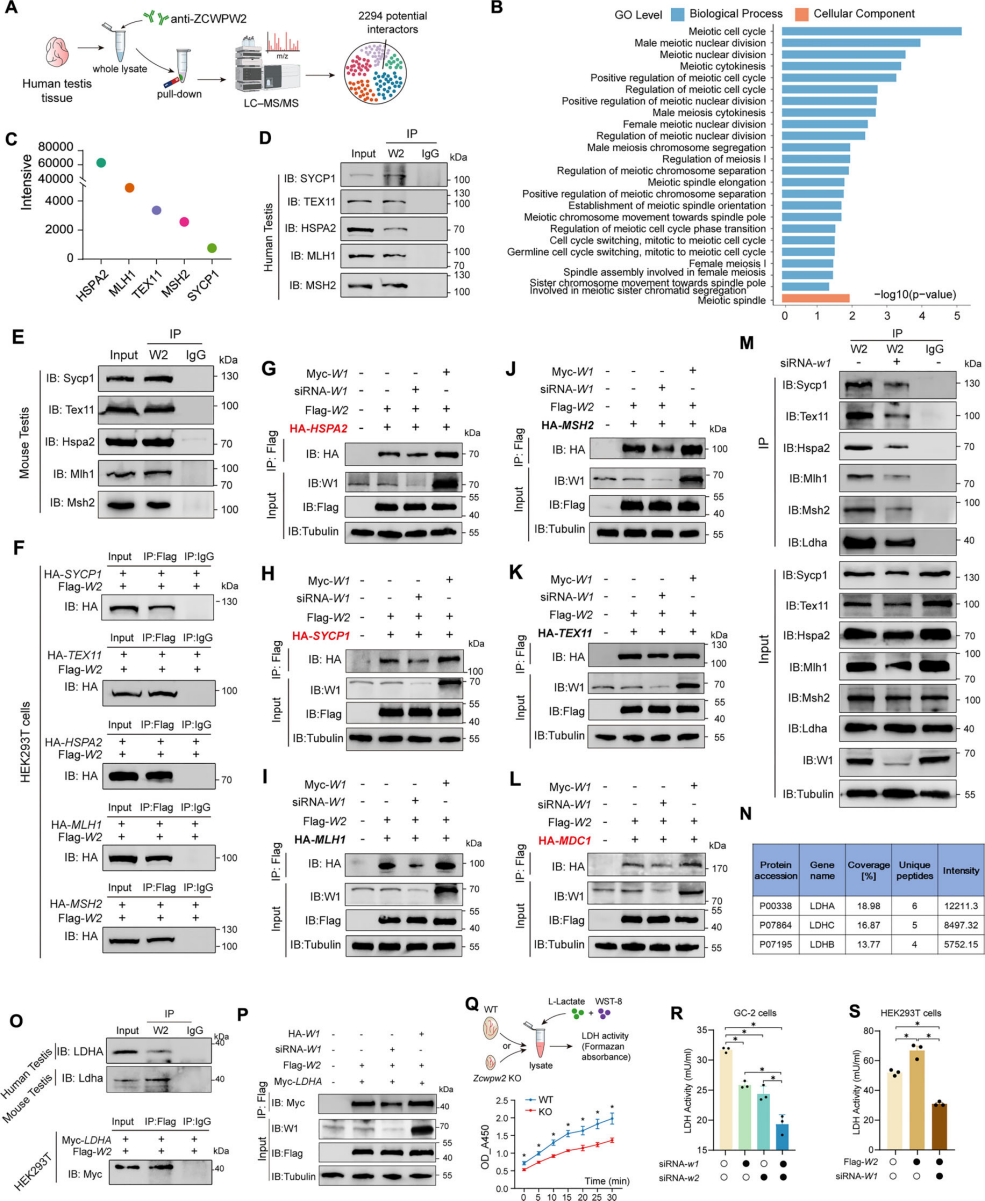
The ZCWPW1–ZCWPW2 complex interacts with recombination-associated proteins and stabilizes LDHA activity. (**A**) Schematic of IP-MS-based identification of ZCWPW2-interacting proteins in human testis tissue. (**B**) GO enrichment analysis of ZCWPW2-interacting proteins. (**C**) Mass spectrometry–based intensity plot depicting meiosis-associated proteins that interact with ZCWPW2. (**D** and **E**) Co-IP analysis of endogenous SYCP1, TEX11, HSPA2, MLH1, and MSH2 proteins interacting with ZCWPW2 from human (D) and mouse (E) testis lysates. (**F**) Co-IP analysis of exogenous SYCP1, TEX11, HSPA2, MLH1, and MSH2 proteins interacting with exogenous ZCWPW2 in HEK293T cells. (**G–L**) Co-IP assays demonstrating the impact of *ZCWPW1* knockdown (siRNA-*W1*) or overexpression (Myc-*W1*) on the interactions of Flag-ZCWPW2 with HA-HSPA2 (G), SYCP1 (H), MLH1 (I), MSH2 (J), TEX11 (K), and MDC1 (L) in HEK293T cells. (**M**) Co-IP assays demonstrating the impact of *Zcwpw1* knockdown (siRNA-*w1*) on the interactions of endogenous Zcwpw2 with Hspa2, Sycp1, Mlh1, Msh2, Tex11, Mdc1, and Ldha in GC-2 cells. (**N**) Table summarizing the LDH family proteins detected in ZCWPW2 immunoprecipitates from human testis by IP-MS. (**O**) Co-IP assays demonstrating the interaction between ZCWPW2 and LDHA in human and mouse testes, as well as transfected HEK293T cells. (**P**) Co-IP analysis examining the interaction between Flag-ZCWPW2 and Myc-LDHA in HEK293T cells under conditions of *ZCWPW1* knockdown (siRNA-*W1*) or overexpression (HA-*W1*). (**Q**) Schematic overview (top) and quantitative analysis (bottom) of LDH enzymatic activity in testis lysates from WT and *Zcwpw2* KO juvenile mice. Activity was measured by assessing formazan absorbance at 450 nm following the conversion of lactate using the WST-8 assay (*n* = 3 biologically independent WT mice and KO mice; two-tailed Student’s *t*-test; *P < 0.05; error bars, s.e.m.). (**R** and **S**) LDH enzymatic activity measured by WST-8-based formazan absorbance in treated GC-2 cells (R) and HEK293T cells (S) (*n* = 3 independent experiments; two-tailed Student’s *t*-test; *P < 0.05; error bars, s.e.m.).

To explore the underlying molecular mechanism of the PRDM9/ZCWPW1/ZCWPW2 system in recombination, we performed IP-MS analysis using human testes to analyze the interacting proteins of ZCWPW2 (Fig. 5A). A total of 2294 proteins were identified, with ZCWPW1 confirmed as an interactor of ZCWPW2. GO analysis revealed that proteins associated with meiosis are significantly enriched (Fig. 5B), particularly those related to the recombination machinery, such as SYCP1, HSPA2, TEX11, MSH2, and MLH1 (Fig. 5C). Further Co-IP validation confirmed the interactions of these proteins with ZCWPW2 in human and mouse testes as well as transfected cells (Fig. 5D–F). In addition, DNase treatment of lysates from testes and cultured cells did not affect the interactions between these proteins and ZCWPW2 (Supplementary Fig. S9A–C).

Given that ZCWPW1 and ZCWPW2 form a complex, we further investigated whether the binding of ZCWPW2 to these proteins depends on ZCWPW1 in cultured cells. To test this, we silenced *ZCWPW1* in HEK293T cells (which express ZCWPW1 but not ZCWPW2) using siRNA and observed a significant reduction in the interactions of exogenous ZCWPW2 with exogenous SYCP1, HSPA2, TEX11, MSH2, and MLH1 (Fig. 5G–L). Conversely, overexpression of ZCWPW1 enhanced these interactions (Fig. 5G–L). Furthermore, in GC-2 cells (a mouse spermatocyte cell line), knockdown of *Zcwpw1* resulted in a similar reduction in the interactions of endogenous ZCWPW2 with endogenous SYCP1, HSPA2, TEX11, MSH2, and MLH1 (Fig. 5M). Therefore, we hypothesized that the ZCWPW1–ZCWPW2 complex might regulate the recombination process through interactions with key proteins involved in the recombination machinery.

Interestingly, we identified 27 ZCWPW2-interacting proteins with potential transcription-promoting functions, 18 of which are histone-modifying enzymes or chromatin remodelers (Supplementary Table S7). Among these histone-modifying enzymes and chromatin remodelers, all except SMARCA2, CHTOP, PRMT1, and NAT10 were enriched at promoter regions. Since ChIP-seq data for ELP3, SMARCD3, RUVBL1, SUPT16H, and ATAD2 were unavailable for our reanalysis, we compared the available ChIP-seq datasets for DOT1L, PHF8, KAT7, SMARCC1, SMARCA5, PARP1, BRD1, RUVBL2, and KDM1A with our CUT&Tag data for ZCWPW2. This comparison revealed a strong overlap between ZCWPW2 peaks and PHF8/RUVBL2 peaks at promoter regions (Supplementary Table S8). PHF8 is a histone demethylase that recognizes the H3K4me3 mark and binds to promoter regions, where it removes H3K9me1/2 and H4K20me1 to activate gene transcription [60]. Moreover, PHF8 is highly expressed in testicular tissue, and a recent study has revealed that it facilitates transcriptional recovery following DSB repair [61]. RUVBL2 is highly expressed in testicular tissue and functions as a component of several ATP-dependent chromatin-remodeling complexes that participate in DNA recombination and repair [62, 63]. Consequently, we validated the interactions of ZCWPW2 with PHF8 and RUVBL2 by Co-IP assay in human and mouse testes (Supplementary Fig. S9D and E). Therefore, we speculated that ZCWPW2 may regulate the transcription of meiosis-related genes and *EP300* through its interactions with PHF8 and RUVBL2.

### The ZCWPW1–ZCWPW2 complex interacts and stabilizes the enzyme activity of lactate dehydrogenase LDHA

Among the ZCWPW2 interactors, the lactate dehydrogenase (LDH) family also caught our attention. Almost all members of this family, including LDHA, LDHB, and LDHC, were significantly enriched, with LDHA showing the highest enrichment as a key protein involved in lactylation (Fig. 5N) [64]. Co-IP assays provided further evidence for the interaction between ZCWPW2 and LDHA in both human and mouse testicular tissues, as well as in transfected cells (Fig. 5O). Similarly, in HEK293T and GC-2 cells, the binding of LDHA to ZCWPW2 was also shown to be dependent on ZCWPW1 (Fig. 5P and M).

Although we found that LDHA is an interactor of ZCWPW2 dependent on ZCWPW1, its abundance was comparable between *Zcwpw2* KO testes and WT testes (Supplementary Fig. S10A). Surprisingly, LDHA enzymatic activity was significantly reduced in *Zcwpw2* KO testes compared with WT testes (Fig. 5Q). Consistently, knockdown of *Zcwpw1* or *Zcwpw2* in GC-2 cells had no impact on LDHA abundance, but its enzymatic activity was markedly reduced compared with control cells, accompanied by a decrease in cytosolic lactate levels (Fig. 5R, and Supplementary Fig. S10B and C). Additionally, overexpression of ZCWPW2 in HEK293T cells did not alter LDHA abundance compared with control cells (Supplementary Fig. S10D), but it increased endogenous LDHA enzymatic activity (Fig. 5S), leading to a corresponding rise in cytosolic lactate levels (Supplementary Fig. S10E). As expected, silencing ZCWPW1 significantly reduced both LDHA enzymatic activity and cytosolic lactate levels (Fig. 5S and Supplementary Fig. S10E). AlphaFold3 further predicted that residues K81, Q101, E102, G103, S105, R112, K118, N123, Y127, D141, H193, D195, Y239, K278, E329, and Q331 of LDHA interact with the ZCWPW1/ZCWPW2 complex. Notably, among these residues, H193 has been reported to be indispensable for LDHA enzymatic activity [65, 66] (Supplementary Fig. S10F). In addition, several other residues (Q101, E102, G103, and S105) are located near R106 (Supplementary Fig. S10F), another residue critical for LDHA activity [65, 66]. Therefore, these findings suggested that the ZCWPW1–ZCWPW2 complex may support LDHA enzymatic activity through their interaction.

### Downregulation of lactylation and abundance of recombination-associated proteins in ZCWPW2-deficient testes

**Figure 6.**
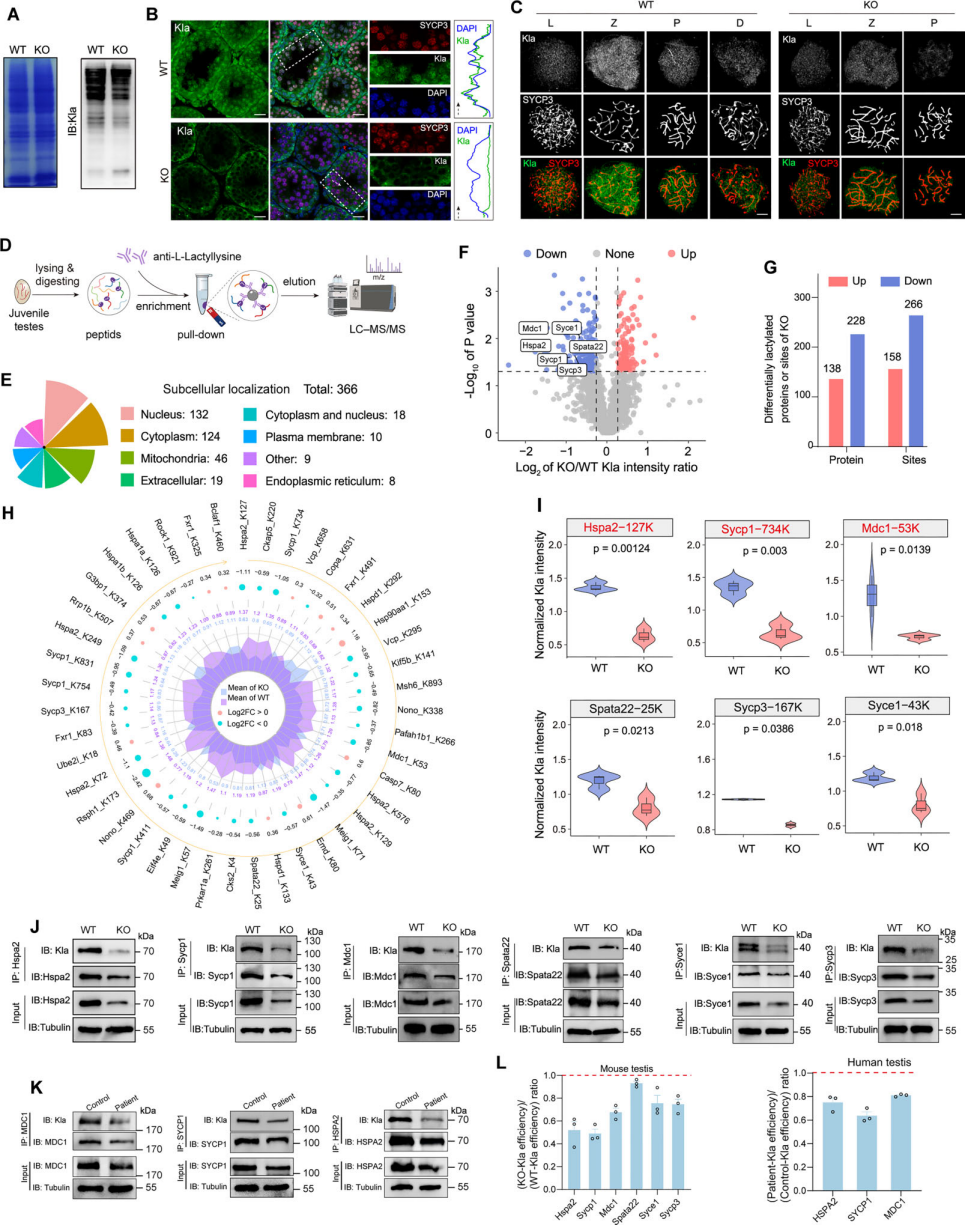
ZCWPW2 deficiency reduces the lactylation and abundance of recombination-related proteins in mouse and patient testes. (**A**) Coomassie brilliant blue staining (left) and immunoblotting (right) of total testicular proteins from WT and *Zcwpw2* KO juvenile mice (postnatal day 12) using a pan anti-lactylation (Kla) antibody to detect global Kla levels. (**B**) Immunofluorescence staining of testis sections from WT and *Zcwpw2* KO juvenile mice using antibodies against pan-Kla (green) and SYCP3 (red), with DAPI counterstaining (blue); scale bar: 50 μm. The dashed arrows indicate the direction and position along which fluorescence intensities were measured. The right panels show representative Kla and DAPI intensity line plots. The white dashed box indicates the magnified image (*n* = 3 biologically independent WT mice and KO mice). (**C**) Immunofluorescence analysis of chromosome spreads from WT and *Zcwpw2* KO spermatocytes stained with Kla (green) and SYCP3 (red). L, Leptotene; Z, Zygotene; P, Pachytene or Pachytene-like; D, Diplotene; scale bars: 5 μm (*n* = 3 biologically independent WT mice and KO mice). (**D**) Schematic representation of the workflow for profiling lactylome in juvenile testes of WT and *Zcwpw2* KO mice (*n* = 3 biologically independent WT mice and KO mice). (**E**) Subcellular localization analysis of Kla-modified proteins identified in juvenile mouse testes. (**F**) Volcano plot showing significantly downregulated (blue) and upregulated (red) Kla proteins in *Zcwpw2* KO versus WT testes. Recombination-related proteins were highlighted. (**G**) Bar graph summarizing the number of differentially expressed Kla proteins (left) and modification sites (right) in KO testes compared to WT testes. (**H**) Circular bar plot showing significantly differentially lactylated proteins between WT and *Zcwpw2* KO testes. Radial bars display the average intensity of Kla-modified peptides in WT (purple) and KO (blue), while colored dots indicate log_2_ fold change (log_2_ FC): red for increased Kla (log_2_ FC > 0) and cyan for decreased Kla (log2 FC < 0) in KO testes. (**I**) Violin plots showing relative Kla levels at specific lysine residues of recombination-associated proteins in WT versus *Zcwpw2* KO testes. (**J**) Co-IP assays validating the reduced Kla and abundance of key recombination-associated proteins—Hspa2, Sycp1, Mdc1, Spata22, Syce1, Sycp3, and Msh6—in testicular tissues from juvenile *Zcwpw2* KO mice compared to WT mice. (**K**) Co-IP analyses confirmed a reduction in Kla and protein abundance of HSPA2, SYCP1, and MDC1 in testicular tissues from the patient carrying the *ZCWPW2* mutation. (**L**) Bar graphs displaying the lactylation efficiency of the recombination-associated proteins in mouse testis and human testicular biopsies. Lactylation efficiency = lactylation signal/protein abundance (quantified by ImageJ). Ratios < 1 indicate reduced lactylation efficiency in KO mice and the patient (*n* = 3 independent experiments; error bars, s.e.m.).

Given our observation that ZCWPW2 participates in mediating *EP300* transcription and that the ZCWPW1–ZCWPW2 complex regulates LDHA enzymatic activity, we speculated that ZCWPW2 may play a role in lactylation regulation during meiosis. As anticipated, post-translational modifications such as acetylation, malonylation, and others exhibited no significant differences between juvenile WT and KO testes (Supplementary Fig. S11A), while lactylation was significantly lower in KO testes than in WT testes (Fig. 6A). We further employed immunofluorescence staining on testes and found that lactylation signals were predominantly elevated in spermatocytes during meiotic prophase in WT mice, while the signals were markedly reduced in KO mice (Fig. 6B). Chromosome spreading experiments further revealed that the lactylation staining was primarily observed in spermatocytes at the zygotene stage in WT mice, whereas almost no detectable signals were present in the KO mice (Fig. 6C and Supplementary Fig. S11B).

We further conducted an analysis of widespread lactylation modifications in the testes of juvenile *Zcwpw2* KO and WT mice (Fig. 6D). A total of 9213 lactylation sites and 2730 proteins were identified in the WT and KO testes (Supplementary Fig. S11C). The number of lactylation sites per protein was similar on each magnitude in the two groups (Supplementary Fig. S11D). We further found that most lactylation sites were located on non-histone proteins in both WT and KO testes, suggesting that non-histone protein lactylation may also play an important role in meiosis (Supplementary Fig. S11E). Besides, the amino acid sequence preference of lactylation was nearly identical between WT and KO testes, with alanine and lysine most frequently occurring near lactylated sites (Supplementary Fig. S11F and G). In addition, cellular compartment analysis revealed that lactylated proteins were mainly localized to the nucleus and cytoplasm, with a slightly higher proportion in the nucleus than in the cytoplasm (Fig. 6E).

Noticeably, the lactylation intensities at many protein sites, reflecting relative lactylation levels, differed significantly, with a greater number of proteins (228) and sites (266) exhibiting a marked decrease in KO testes compared to WT (Fig. 6F and G). Proteins with significantly altered relative lactylation levels are shown in Fig. 6H, with those exhibiting downregulated levels mainly involved in the meiotic process. GO analysis of proteins showing a decrease in relative lactylation levels revealed significant enrichment in spermatogenesis and male gamete generation, particularly in the meiotic process involved in recombination machinery, such as meiotic DNA repair synthesis in the Biological Process category, and in the synaptonemal complex in the Cellular Component category (Supplementary Fig. S11H). Among the downregulated sites (Supplementary Fig. S11I), several sites on recombination-associated proteins exhibited the most significant decreases in relative lactylation levels, including Hspa2–127K (*P* = 0.001), Hspa2–129K (*P* = 0.015), Hspa2–576K (*P* = 0.015), Sycp1–734K (*P* = 0.003), Mdc1–53K (*P* = 0.013), Spata22–25K (*P* = 0.021), Syce1–43K (*P* = 0.018), and Sycp3–167K (*P* = 0.038) (Fig. 6I and Supplementary Fig. S11J). Mass spectrometry data extracted from the raw data confirmed the presence of lactylation at the sites of these key proteins (Supplementary Fig. S12). The high conservation of these sites across diverse species, from river otters and horses to mice, pandas, and humans, indicates their evolutionary stability and essential biological functions (Supplementary Fig. S13A).

Based on the decrease in relative lactylation levels at these sites, we constructed five sets of plasmids, each containing a Flag-tagged wild-type, single-site mutant, and multiple-site mutant version. Compared to the wild-type forms of HSPA2, SYCP1, MDC1, SPATA22, and SYCP3, the single-site variants decreased the lactylation signal on these proteins when co-transfected with *ZCWPW2*, whereas the multiple-site variants resulted in an even greater reduction under the same conditions (Supplementary Fig. S13B–F). As expected, the downregulation of lactylation on these proteins was validated in the testicular tissue of *Zcwpw2* KO mice (Fig. 6J). Importantly, the markedly reduced lactylation of HSPA2, SYCP1, and MDC1 was similarly detected in the patient’s testicular biopsy tissue (these three proteins were selected because no additional testicular tissue was available and they showed the most pronounced downregulation of relative lactylation levels in KO testes) (Fig. 6K). Interestingly, we found that the abundance of these proteins with downregulated lactylation was also reduced in the testes of KO mice and the patient, as well as in transfected cells overexpressing plasmids with mutant lactylation sites (Fig. 6J and K, and Supplementary Fig. S13B–F).

To clarify that the downregulated lactylation was not a consequence of decreased protein abundance, we quantified both the lactylation signal and the corresponding immunoprecipitated protein signal to calculate the lactylation efficiency in WT and mutant samples [43, 44]. The results revealed that the lactylation efficiency was consistently lower in the mutant group compared with the WT group (ratio <1) (Fig. 6L). Therefore, we speculated that ZCWPW2 might mediate the lactylation of recombination-related proteins and maintain their abundance, thereby contributing to the recombination process.

### The ZCWPW1–ZCWPW2 complex stabilizes recombination-related proteins via LDHA/EP300-mediated lactylation in cultured cells

**Figure 7.**
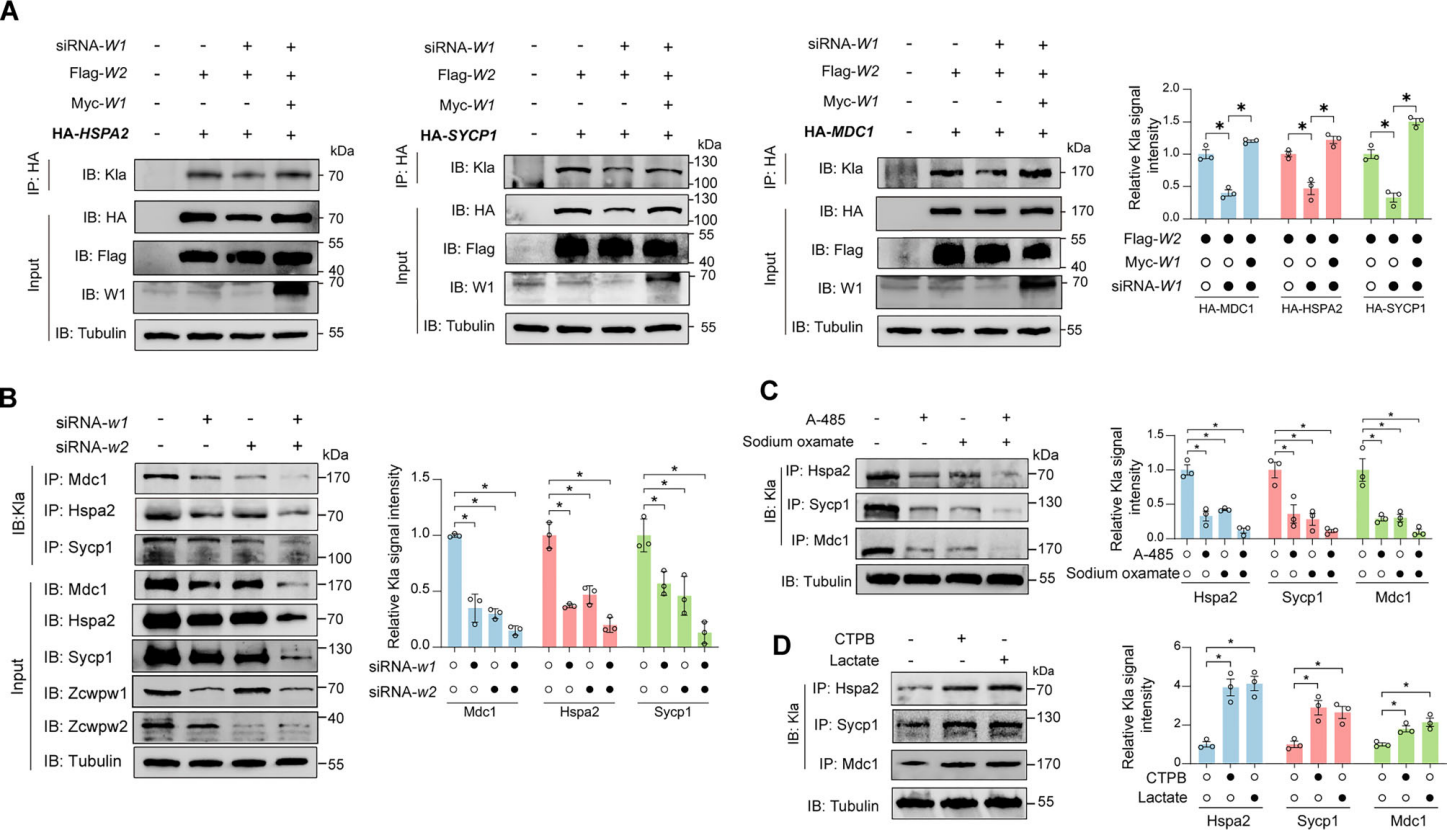
ZCWPW2 promotes lactylation and stability of recombination-associated proteins through coordination with ZCWPW1 in cultured cells. (**A**) Co-IP analysis of HEK293T cells overexpressed with HA-recombination-associated proteins (HSPA2, SYCP1, and MDC1) and Flag-ZCWPW2, with or without Myc-ZCWPW1, and in the presence or absence of siRNA targeting *ZCWPW1*. Bar graph quantifies Kla signal intensity for each condition (*n* = 3 independent experiments; two-tailed Student’s *t*-test; *P < 0.05; error bars, s.e.m.). (**B**) Co-IP analysis (left) for Kla of endogenous Mdc1, Hspa2, and Sycp1 in GC-2 cells after individual or combined knockdown of *Zcwpw1* or/and *Zcwpw2* using siRNAs. Quantification of Kla levels (right) showed a significant decrease in Kla upon *Zcwpw1* or *Zcwpw2* knockdown, with the greatest reduction observed upon dual silencing (*n* = 3 independent experiments; two-tailed Student’s *t*-test; *P < 0.05; error bars, s.e.m.). (**C** and **D**) Co-IP (left) and quantification analysis (right) of Kla on endogenous Mdc1, Hspa2, and Sycp1 in GC-2 cells treated with the EP300 inhibitor A-485, the LDHA inhibitor sodium oxamate, or both (C), and with EP300 activator CTPB or exogenous lactate (D) (*n* = 3 independent experiments; two-tailed Student’s *t*-test; *P < 0.05; error bars, s.e.m.).

To further confirm that the ZCWPW1–ZCWPW2 complex promotes lactylation of recombination machinery–associated proteins and further stabilizes them, HEK293T cells were co-transfected with *ZCWPW2* and *SYCP1, HSPA2*, or *MDC1* plasmids (Fig. 7A). As expected, knockdown of ZCWPW1 led to reduced lactylation levels of SYCP1, HSPA2, and MDC1 compared to control cells, accompanied by a decrease in their abundance (Fig. 7A). Notably, overexpression of ZCWPW1 effectively restored both the lactylation levels and the abundance of these proteins (Fig. 7A).

Furthermore, we obtained comparable results in GC-2 cells, where silencing of *Zcwpw1* reduced the lactylation and protein levels of endogenous Sycp1, Hspa2, and Mdc1 (Fig. 7B). Next, we knocked down *Zcwpw2* in GC-2 cells and similarly observed a significant downregulation in the lactylation and protein levels of these proteins (Fig. 7B). As expected, simultaneous silencing of *Zcwpw1* and *Zcwpw2* in GC-2 cells led to a further decrease in the lactylation and protein levels of these proteins (Fig. 7B).

Since ZCWPW2 promotes protein lactylation by stabilizing the function of the key lactylation enzymes EP300 and LDHA, we next examined whether inhibition of these enzymes would influence the lactylation and protein abundance of recombination-associated proteins. Notably, the lactylation levels and protein abundance of both endogenous and exogenous SYCP1, HSPA2, and MDC1 were significantly reduced upon treatment with the EP300 inhibitor A-485 or the LDHA inhibitor sodium oxamate in GC-2 and HEK293T cells (Fig. 7C and Supplementary Fig. S14A). Furthermore, a more pronounced reduction in lactylation levels and protein abundance of these proteins was observed when both EP300 and LDHA inhibitors were applied, compared with cells treated with either inhibitor alone (Fig. 7C and Supplementary Fig. S14A). In contrast, treatment with an EP300 activator CTPB or with lactate markedly increased the lactylation levels and protein abundance of both exogenous and endogenous SYCP1, HSPA2, and MDC1 compared with control cells (Fig. 7D and Supplementary Fig. S14B).

Lactylation has been suggested to suppress ubiquitination-mediated protein degradation, thereby stabilizing protein abundance [67–69]. Interestingly, knockdown of *Zcwpw1* or *Zcwpw2* in GC-2 cells led to a marked increase in the ubiquitination of endogenous Sycp1, Hspa2, and Mdc1 compared with control cells (Supplementary Fig. S14C). As expected, increased ubiquitination of Sycp1, Hspa2, and Mdc1 was detected in *Zcwpw2*-deficient testes (Supplementary Fig. S14D). These results suggested that ZCWPW1–ZCWPW2 may promote lactylation to prevent ubiquitination-mediated protein degradation.

**Figure 8.**
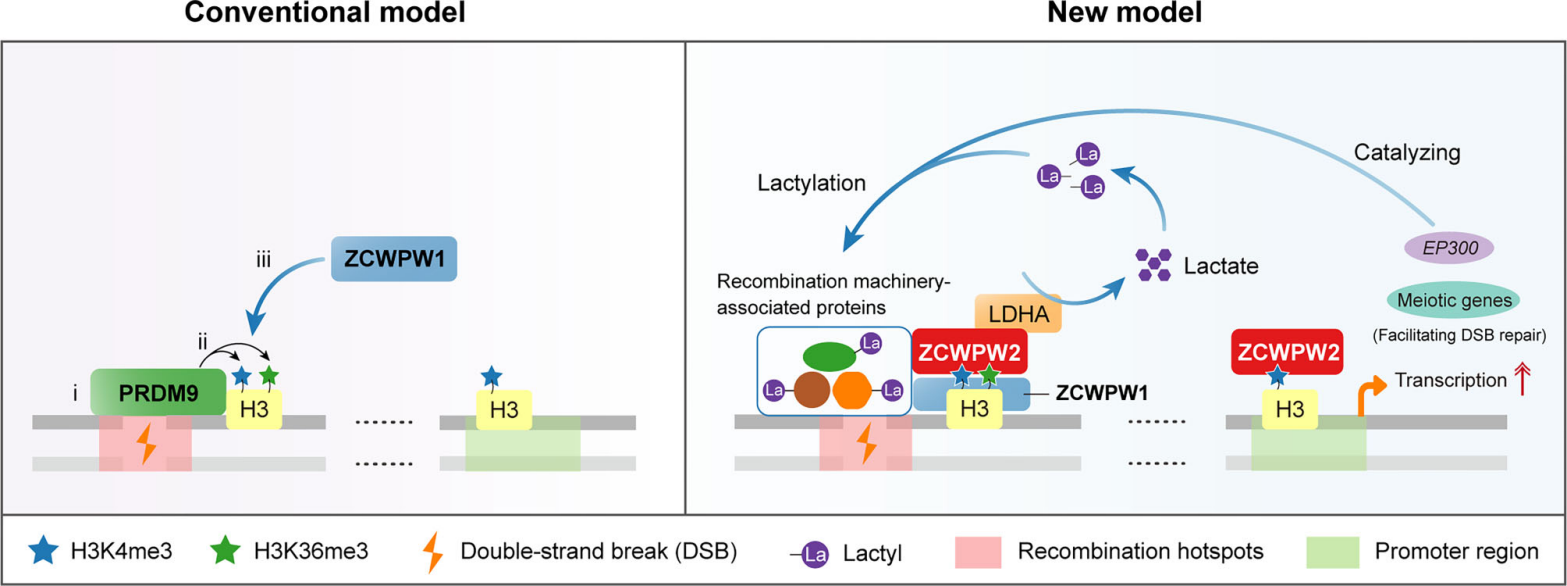
Proposed schema of PRMD9–ZCWPW1–ZCWPW2 system regulating recombination progression. During meiotic recombination, PRDM9 marks the dual histone methylation of H3K4me3 and H3K36me3 at nucleosomes to define recombination hotspots (i, ii), while ZCWPW1 acts as a reader by recognizing these modifications (iii). In this study, we revealed that ZCWPW2 shows enhanced enrichment at dual histone methylation sites in the presence of PRDM9 and forms a complex with ZCWPW1 to interact with recombination machinery-associated proteins at these sites. Additionally, the ZCWPW1–ZCWPW2 complex interacts with and stabilizes the enzymatic activity of lactate dehydrogenase LDHA, promoting local lactate production. In parallel, *ZCWPW2* binds independently to H3K4me3-rich promoter regions, regulating the transcription of meiosis-related genes, as well as that of the lactylation writer EP300 to catalyze lactylation. These two coordinated actions further stabilize the recombination machinery via the lactylation pathway, thereby successfully facilitating DSB repair during recombination progression.

In summary, the ZCWPW1–ZCWPW2 complex interacts with and stabilizes LDHA activity, while ZCWPW2 enhances EP300 transcription, together promoting the lactylation of recombination machinery–associated proteins and thereby stabilizing their abundance by preventing ubiquitination-mediated degradation (Fig. 8).

## Discussion

During the recombination process, PRDM9 first deposits both H3K4me3 and H3K36me3 at its binding sites, and this action is crucial for the positioning of recombination hotspots [7]. Subsequently, ZCWPW1 localizes to recombination hotspots by recognizing the dual histone marks deposited by PRDM9 [29]. However, it remains unclear whether additional members are involved in this system and how it regulates the recombination process. ZCWPW2, a paralog of ZCWPW1 containing both zf-CW and PWWP domains, binds to H3K4me3 and H3K36me3 *in vitro*, with stronger binding when both marks are present [33, 34]. We therefore speculated that ZCWPW2 may function as a component of the PRDM9 system. However, currently available commercial antibodies for ZCWPW2 are not suitable for chromatin immunoprecipitation techniques such as ChIP or CUT&Tag. Importantly, a previous study performed ChIP-seq analysis on HEK293T cells transfected with human *PRDM9* (*PRDM9*^B^ variant) plasmid and compared the resulting PRDM9 peaks with human recombination hotspots from DMC1 ChIP-seq in testes, showing that PRDM9 binds most hotspots marked by H3K4me3 and H3K36me3 [49]. Building on this, they transfected HEK293T cells with full-length HA-tagged human *ZCWPW1*, alone or with full-length human *PRDM9^B^*, and found that in the presence of PRDM9, ZCWPW1 was strongly enriched at PRDM9-binding sites, likely through recognition of the dual H3K4me3–H3K36me3 mark [29]. These findings agree with results in testicular tissue and spermatocytes, where ZCWPW1 binding is promoted by PRDM9 in an allele-specific manner [30, 31]. Thus, PRDM9 overexpression in HEK293T cells is sufficient to establish H3K4me3 and H3K36me3 at hotspots. Based on this, we performed CUT&Tag analysis using an anti-Flag antibody in HEK293T cells transfected with full-length human Flag-tagged *ZCWPW2*, either alone or co-transfected with human Myc-tagged *PRDM9^B^*. We observed that, in the presence of PRDM9, ZCWPW2 peaks were significantly more enriched at PRDM9 sites than in its absence. In addition, in the presence of PRDM9, ZCWPW2 peaks were much more enriched at sites marked by both H3K4me3 and H3K36me3 compared with the absence of PRDM9, showing nearly a twofold increase. Furthermore, our findings revealed that ZCWPW1 and ZCWPW2 form a complex at these dual histone methylation sites. Through IP-MS analysis, ZCWPW2 was found to interact with recombination machinery-associated proteins, including SYCP1, HSPA2, TEX11, MSH2, and MLH1. Further investigation revealed that the binding of ZCWPW2 to these proteins was dependent on ZCWPW1. Therefore, in the presence of PRDM9, ZCWPW2 shows increased enrichment at the dual histone methylation sites and associates with ZCWPW1 to interact with recombination machinery-associated proteins at these sites.

Our findings, together with previous studies, highlight the essential role of the PRDM9–ZCWPW1–ZCWPW2 system in meiotic recombination. Although the loss of each of these three proteins causes defects in homologous chromosome synapsis and DSB repair, their functions are not completely identical. In the absence of ZCWPW1 [32] or ZCWPW2 (as shown in our study), recombination hotspots are unaffected and remain positioned within the dual histone marks of H3K4me3 and H3K36me3 catalyzed by PRDM9. However, in the absence of PRDM9, recombination hotspots are not eliminated but instead shift predominantly to promoters and other genomic regions marked by PRDM9-independent H3K4me3 [45]. These observations are consistent with the role of PRDM9 as a “writer” in the recombination process, whereas ZCWPW1 and ZCWPW2 function as “readers” and play subsequent regulatory roles in the recruitment of the recombination machinery. The *Prdm9*-deficient female mice lack pachytene oocytes from very early developmental stages, and males exhibit complete spermatocyte arrest [45]. We found that mice lacking ZCWPW2 exhibit a similar phenotype in both sexes. However, *Zcwpw1* KO females exhibit normal fertility at a young age (8 weeks) but display a marked delay in meiotic progression [32]. By late adulthood (around 8 months), they become completely infertile, characterized by premature ovarian insufficiency [32]. Although *Zcwpw1* KO males are completely infertile [32], their homologous chromosome synapsis defects are less severe than those observed in *Zcwpw2* KO males. Consistently, we found that our *ZCWPW2*-mutant patient exhibited more pronounced meiotic arrest than a reported *ZCWPW1*-mutant patient, with the *ZCWPW2*-mutant testis containing only spermatogonia and spermatocytes, whereas the *ZCWPW1*-mutant testis still showed residual sperm production [48]. Importantly, our CUT&Tag analysis revealed that, in addition to its shared role with ZCWPW1 at the dual histone methylation sites, ZCWPW2 uniquely binds to H3K4me3-rich promoter regions independent of PRDM9. Moreover, most of the genes in these promoter regions are related to the meiotic process, and ZCWPW2 may promote their transcription through its interaction with PHF8 and RUVBL2. This may explain why ZCWPW2 deficiency leads to a more severe phenotype than ZCWPW1 deficiency. Certainly, the phenotypic similarities and differences among the three strains are closely related to the distinct roles each play in the recombination process. Moreover, we detected moderate abundance of ZCWPW2 in post-meiotic germ cells, suggesting a potential role in sperm morphogenesis. This warrants further investigation using conditional KO of *Zcwpw2* in secondary spermatocytes.

Epigenetic modifications, such as histone methylation and acetylation, as well as post-translational phosphorylation, are known to play important roles in meiotic regulation [70]. However, the role of lactylation in meiosis remains largely unknown. Lactylation is a novel modification involving the covalent attachment of a lactyl group to lysine residues, broadly distributed in histone and non-histone proteins and implicated in gene regulation under both physiological and pathological conditions [71, 72]. Interestingly, Lin *et al.* discovered a link between lactylation and meiosis in mouse oocytes, showing that abnormal histone lactylation levels, including Pan lactylation, H3K18la, and H4K12la, ultimately disrupt meiotic progression [73]. Lactate, a metabolic byproduct of glycolysis, serves as the substrate for lactylation and facilitates this modification through lactyl group donors such as lactyl-CoA. In fact, male germ cells rely on lactate for their development and function, and lactate deficiency is associated with impaired fertility or loss of spermatocytes in mice, rats, and humans, suggesting that lactylation may play an important role in spermatogenesis [74–76]. During our study, a recent report suggested that H4K8la is closely associated with recombination hotspots, particularly through colocalization and interaction with PRDM9, and further showed that the lactyltransferase HBO1 can induce H4K8la, while pharmacological inhibition of HBO1 in mice reduces H4K8la levels and disrupts meiosis [77]. Moreover, several recent studies have highlighted the pivotal role of protein lactylation in regulating DNA recombination repair during tumorigenesis [78–80]. In the present study, we found that the ZCWPW1–ZCWPW2 complex interacts with LDHA, a lactate dehydrogenase crucial for lactylation [64], and maintains its enzymatic activity. It has been reported that mouse testicular organ cultures treated with an LDHA inhibitor exhibit a meiotic deficiency phenotype [77]. In addition to LDHA, we found that ZCWPW2 promotes the transcription of *EP300*, which has been identified as a key lactylation writer [81]. These findings indicated that lactylation regulation may be an important function of ZCWPW2 during the recombination process. As expected, quantitative lactylome profiling revealed a marked reduction in global lactylation levels in *Zcwpw2* KO testes, particularly in meiotic recombination-related proteins such as Hspa2–127K, Hspa2–129K, Hspa2–576K, Spata22–25K, Syce1–43K, Sycp1–734K, Sycp3–167K, and Mdc1–53K. We further demonstrated that the downregulation of lactylation in these recombination-related proteins resulted in reduced protein abundance in ZCWPW2-deficient testes and cultured cells, as the diminished lactylation failed to prevent the ubiquitin-mediated protein degradation. Therefore, we suggested that the ZCWPW1–ZCWPW2 complex interacts with LDHA to sustain its enzymatic activity, together with ZCWPW2-mediated transcriptional regulation of *EP300*, thereby facilitating lactylation-dependent stabilization of the essential recombination proteins and ensuring the proper execution of meiotic recombination events. Meanwhile, our study is the first to reveal the essential role of non-histone protein lactylation in meiosis. As the previous study has suggested that H4K8la plays an important role in meiotic recombination [77], we examined whether the H4K8la levels were altered in our *Zcwpw2* KO mice compared with WT controls. However, we observed no obvious differences in H4K8la signals between WT and *Zcwpw2* KO testes or in meiotic prophase spermatocytes, including at the zygotene stage where H4K8la expression peaks (Supplementary Fig. S15). A possible explanation is that the earlier study reported interactions between H4K8la and PRDM9 at recombination hotspots, suggesting that H4K8la may cooperate with PRDM9 in defining recombination hotspot locations [77]. Since recombination hotspots are not disrupted in *Zcwpw2* KO mice, the unchanged H4K8la expression supports the idea that ZCWPW2 may function downstream of H4K8la.

In conclusion, our findings revealed a critical role for ZCWPW2 in regulating meiotic recombination in both humans and mice. ZCWPW2 is enriched at histone sites co-marked by H3K4me3 and H3K36me3 in the presence of PRDM9, and independently functions as a meiotic transcriptional modulator in mammals. Together with the elucidated molecular mechanisms by which the ZCWPW1–ZCWPW2 complex regulates the recombination process, our findings highlighted an essential role of the histone methylation writer–reader system in orchestrating the complex developmental program of gametogenesis.

## Supplementary Material

gkag049_Supplemental_Files

## Data Availability

All the necessary data to assess the conclusions in this paper are available in both the main document and the Supplementary Material. We deposited all relevant raw data into public repositories, including the ChIP-seq raw data (accession number: HRA015008, https://ngdc.cncb.ac.cn/gsa-human/browse/HRA015008), IP-MS data (accession number: PXD070427, https://www.ebi.ac.uk/pride/archive/projects/PXD070427), and the lactylomic proteomics data (accession number: PXD068838, https://www.ebi.ac.uk/pride/archive/projects/PXD068838).
