# Supplementary material for: A novel dual histone mark reader ZCWPW2 regulates meiotic recombination through lactylation and transcriptional regulation in humans and mice
Source: Nucleic Acids Res. 2026 Jan 23;54(3):gkag049. doi: 10.1093/nar/gkag049 (PMC12828234; doi:10.1093/nar/gkag049)
Supplement: gkag049_Supplemental_Files [file gkag049_supplemental_files.zip › Supplementary information.pdf]

.

Supplementary Materials for

A novel dual histone mark reader ZCWPW2 regulates meiotic recombination through lactylation and transcriptional regulation in humans and mice

Tiechao Ruan *et al*

*Corresponding author Email: mudz@scu.edu.cn; wanglingbo@fudan.edu.cn; shenying01@scu.edu.cn.

This PDF file includes:

Figure. S1 to S15

Table. S1 to S5; Table S8

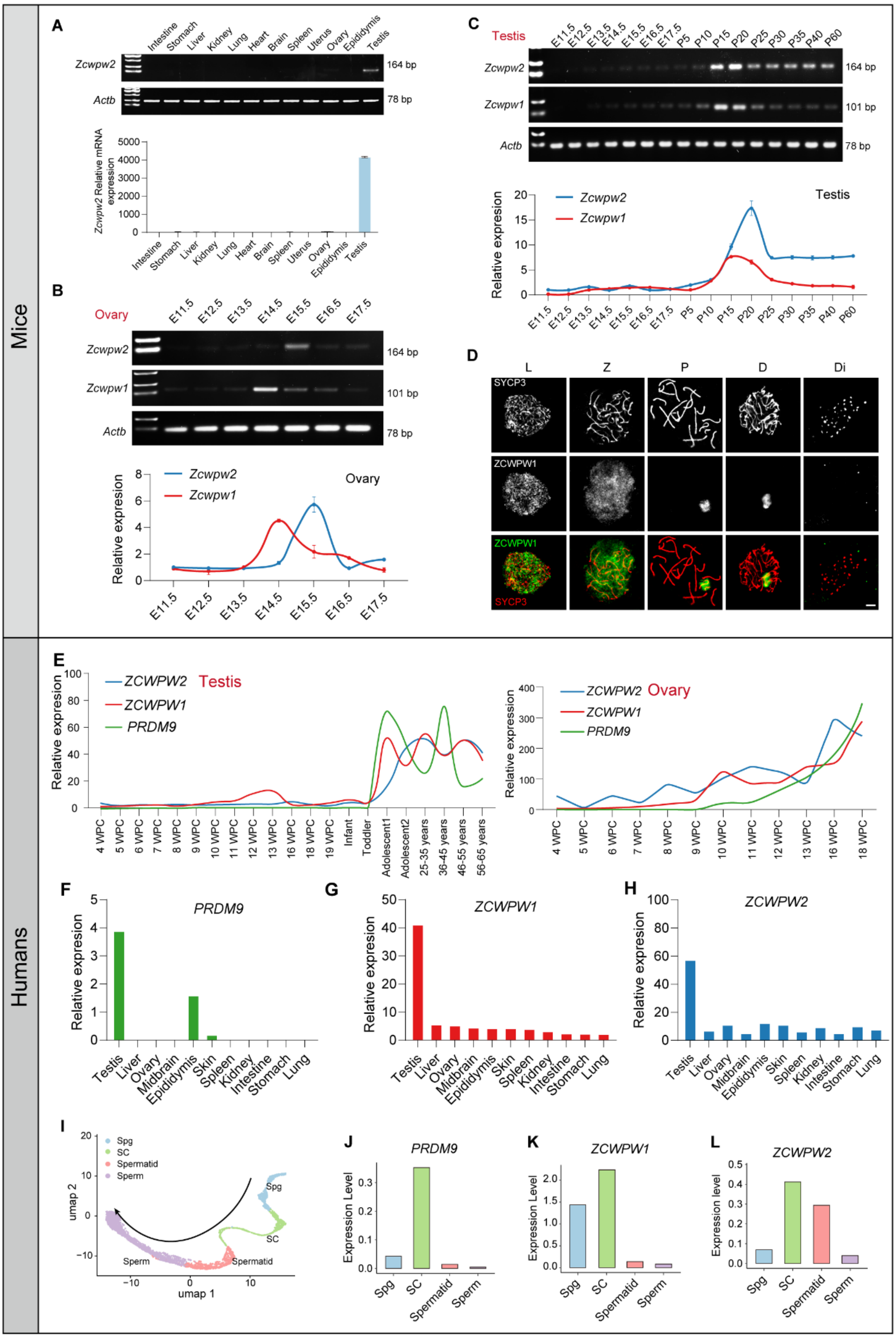

Fig. S1 Expression dynamics of ZCWPW1, ZCWPW2, and PRDM9 during germ cell development in mice and humans. (A) RT-PCR (top) and quantitative analysis

(bottom) of *Zcwpw2* mRNA levels across various adult mouse tissues, normalized to *Actb* expression. (n = 3 independent experiments; error bars, s.e.m.). **(B)** RT-PCR (top) and quantitative analysis (bottom) showing dynamic changes in *Zcwpw2* and *Zcwpw1* expression in embryonic ovary from E11.5 to E17.5. E, embryonic day. (n = 3 independent experiments; error bars, s.e.m.). **(C)** RT-PCR analysis (top) and quantitative expression profiling (bottom) of *Zcwpw2* and *Zcwpw1* in testis across embryonic days (E11.5 to E17.5) and postnatal stages (P5 to P60). P, postnatal day. (n = 3 independent experiments; error bars, s.e.m.). **(D)** Immunofluorescence staining of ZCWPW1 (green) and SYCP3 (red) of chromosome spreads from mouse spermatocytes at different meiotic substages. L, Leptotene; Z, zygotene; P, pachytene; D, diplotene; and Di, diakinesis. Scale bar, 5 μm. **(E)** Line graph showing the relative expression patterns of *PRDM9* (green), *ZCWPW1* (red), and *ZCWPW2* (blue) across various stages of human testicular (right) and ovarian development (left). WPC, weeks post-conception. Data were obtained from the MeDAS database (https://das.chenlulab.com/). **(F-H)** Bar graphs showing relative mRNA expression levels of *PRDM9* (F), *ZCWPW1* (G), and *ZCWPW2* (H) across multiple adult human tissues. Data were derived from the Human Protein Atlas (https://www.proteinatlas.org). **(I)** UMAP plot displaying the trajectory of spermatogenesis and clustering of cell types based on transcriptional profiles. Spg, spermatogonia; SC, Spermatocytes. Data were obtained from single-cell RNA-seq analysis of human testicular cells (GEO accession: GSE112013). **(J-L)** Bar plots showing the expression levels of *PRDM9* (J), *ZCWPW1* (K), and *ZCWPW2* (L) across

distinct spermatogenic cell populations, including spermatogonia (Spg), spermatocytes (SC), spermatids, and sperm. Data were obtained from single-cell RNA-seq analysis of human testicular cells (GEO accession: GSE112013).

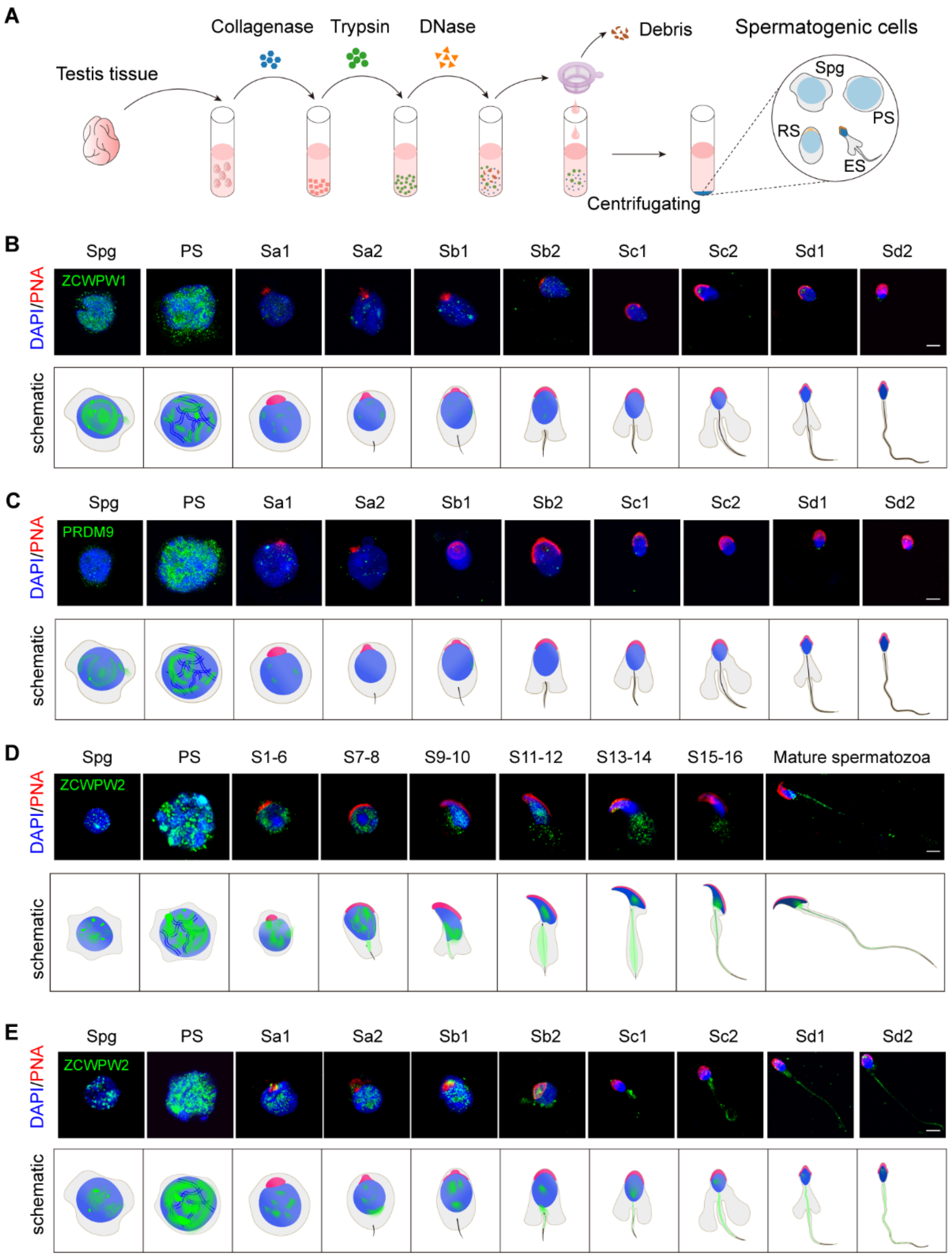

Fig. S2 The expression and localization of ZCWPW1, PRDM9, and ZCWPW2 across spermatogenic stages in humans and mice. (A) Schematic illustration of the protocol used to isolate spermatogenic cells from human testicular biopsies of patients with obstructive azoospermia and from WT mouse testes. **(B and C)**

Immunofluorescence staining of ZCWPW1 (B) and PRDM9 (C) across spermatogenic stages in human germ cells. Scale bar, 5 μm. DAPI (blue) marks nuclei, PNA (red) labels the acrosome, and green signals indicate ZCWPW1 or PRDM9. Schematic illustrations below each image depicted key structural and stage-specific expression patterns. **(D and E)** Immunofluorescence staining of ZCWPW2 across spermatogenic stages in mouse (D) and human (E) germ cells. Scale bar, 5 μm. DAPI (blue) marks nuclei, PNA (red) labels the acrosome, and green indicates ZCWPW2 localization. Schematic illustrations below depicted the expression and location of ZCWPW2 and representative cellular morphologies at each stage.

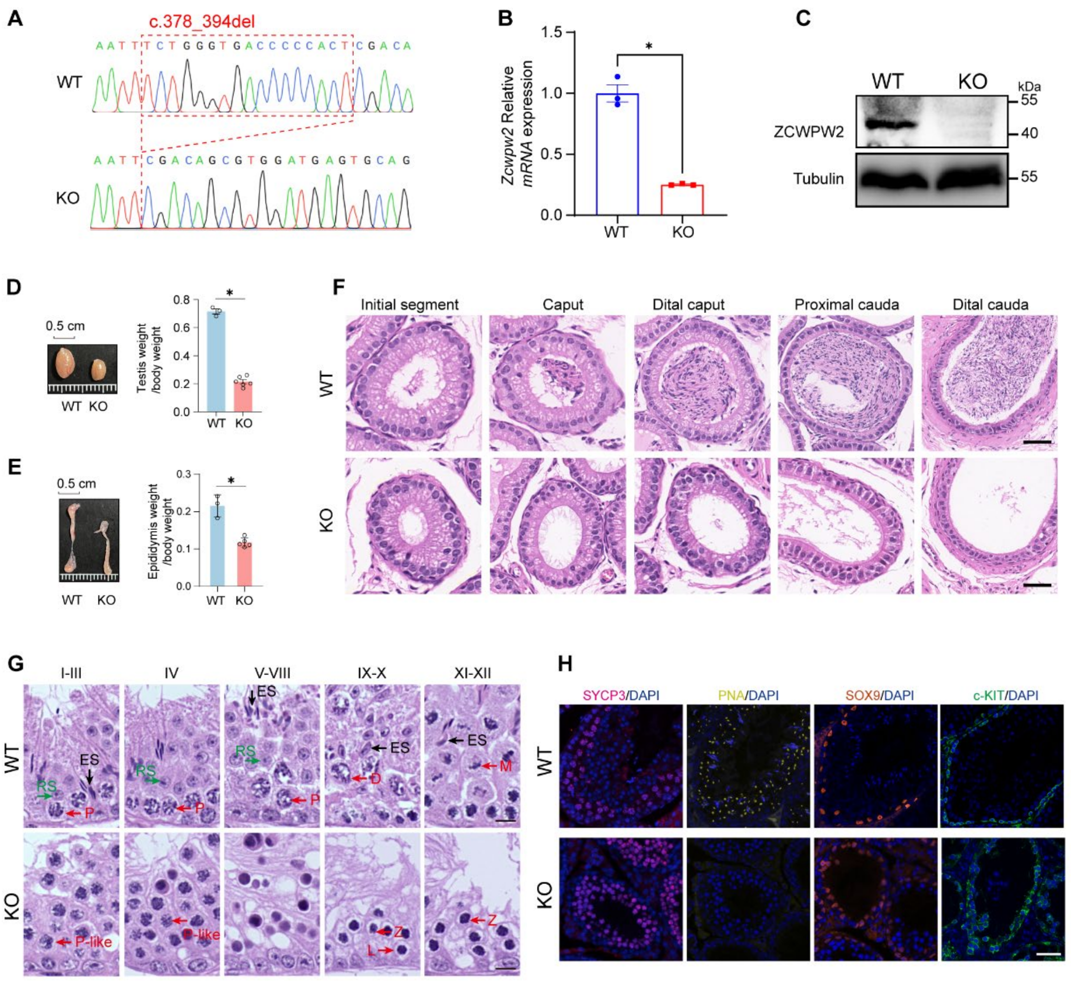

Fig. S3 Generation and the infertile phenotype of *Zcwpw2* KO mice. (A) Sanger sequencing confirming the c.378_394del mutation in *Zcwpw2* introduced by CRISPR/Cas9-mediated genome editing in KO mice. **(B)** qPCR analysis showing significantly reduced *Zcwpw2* mRNA levels in testes of KO mice compared to WT mice, normalized to *Actb*. (n = 3 biologically independent WT mice and KO mice; Two-tailed Student's t test; *p < 0.05; error bars, s.e.m.). **(C)** Immunoblotting of testicular lysates confirmed loss of ZCWPW2 protein in KO mice. **(D)** Representative images of testes from adult WT and *Zcwpw2* KO male mice (left), and quantification of testis weight relative to body weight (right). (n = 3 biologically independent WT mice and KO mice; Two-tailed Student's t test; *p < 0.05; error bars, s.e.m.). **(E)**

Representative images of epididymides from adult WT and KO male mice (left), and quantification of epididymal weight relative to body weight (right). (n = 3 biologically independent WT mice and KO mice; Two-tailed Student's t test; $*p < 0.05$; error bars, s.e.m.). **(F)** H&E staining of epididymal sections from adult WT and KO mice, showing the initial segment, caput, distal caput, proximal cauda, and distal cauda. Scale bars, 50 µm. **(G)** H&E staining of testis sections from adult WT and *Zcwpw2* KO male mice at seminiferous epithelial stages I–XII. P, Pachytene spermatocytes; D, Diplotene spermatocytes; M, Meiotic metaphase spermatocytes; RS, Round spermatids; ES, Elongated/elongating spermatids. Scale bar, 20 µm. **(H)** Immunofluorescence staining of testis sections from adult WT and *Zcwpw2* KO mice using SYCP3 (magenta), PNA (yellow), SOX9 (orange), and c-KIT (green), with DAPI counterstaining (blue). Scale bar, 50 µm.

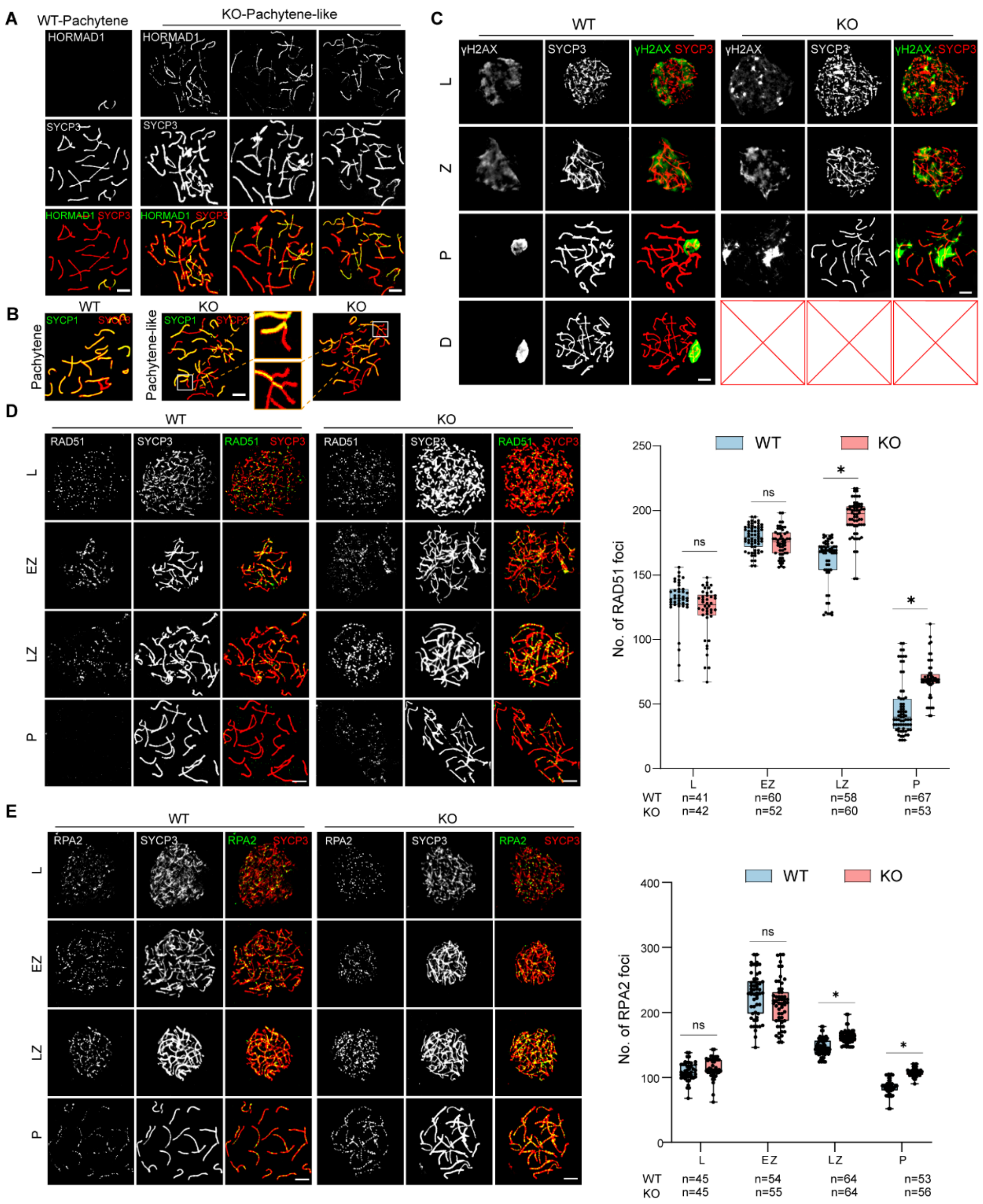

Fig. S4 ZCWPW2 deficiency disrupts DSB repair and synapsis in spermatocytes.

(A) Immunofluorescence staining of chromosome spreads from WT pachytene and *Zcwpw2* KO pachytene-like spermatocytes using antibodies against HORMAD1 (green) and SYCP3 (red). Scale bars, 5 μm. **(B)** Immunofluorescence staining of for SYCP1 (green) and SYCP3 (red) in chromosome spreads from WT and *Zcwpw2* KO pachytene spermatocytes. The enlarged box highlights non-homologous chromosome

synapsis. Scale bar, 5 μm. **(C)** Immunofluorescence staining of γH2AX (green) and SYCP3 (red) in chromosome spreads from WT and *Zcwpw2* KO spermatocytes. L, Leptotene; Z, Zygotene; P, Pachytene or Pachytene-like; D, Diplotene. Scale bar, 5 μm. **(D)** Immunofluorescence staining of RAD51 (green) and SYCP3 (red) on chromosome spreads from WT and *Zcwpw2* KO spermatocytes across different meiotic substages. L, Leptotene; EZ, Early zygotene; LZ, Late zygotene; P, Pachytene or Pachytene-like. Scale bars, 5 μm. n, the number of spermatocytes. (Two-tailed Student's t test; $*p < 0.05$; ns, no significance; error bars, s.e.m.). **(E)** Immunofluorescence staining of RPA2 (green) and SYCP3 (red) across different meiotic substages. Scale bars, 5 μm. L, Leptotene; EZ, Early zygotene; LZ, Late zygotene; P, Pachytene or Pachytene-like. n, the number of spermatocytes. (Two-tailed Student's t test; $*p < 0.05$; ns, no significance; error bars, s.e.m.).

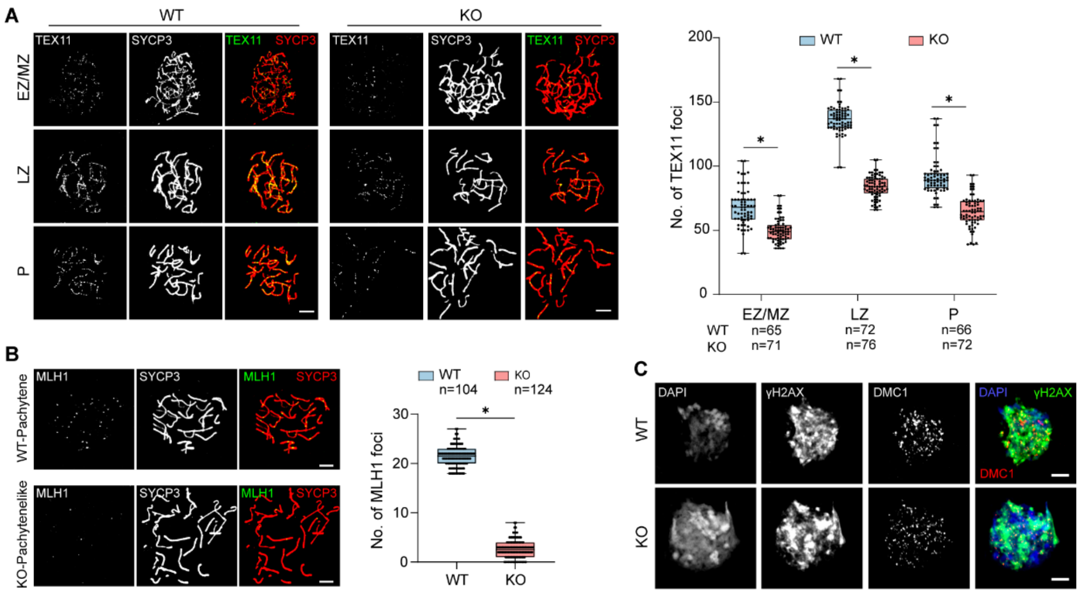

Fig. S5 ZCWPW2 deficiency impairs recombination and crossover in spermatocytes. (A) Immunofluorescence staining of TEX11 (green) and SYCP3 (red) on chromosome spreads from WT and *Zcwpw2* KO spermatocytes at early/mid zygotene (EZ/MZ), late zygotene (LZ), and pachytene or pachytene-like (P) substages. Scale bars, 5 µm. n, the number of spermatocytes. (Two-tailed Student's t test; $*p < 0.05$; error bars, s.e.m.). **(B)** Immunofluorescence staining of chromosome spreads from WT and *Zcwpw2* KO pachytene or pachytene-like spermatocytes using antibodies against MLH1 (green) and SYCP3 (red). n, the number of spermatocytes. (Two-tailed Student's t test; $*p < 0.05$; error bars, s.e.m.). **(C)** Immunofluorescence staining of DMC1 (red), γH2AX (green), and DAPI (blue) in chromosome spreads from WT and *Zcwpw2* KO spermatocytes. Scale bar, 5 µm.

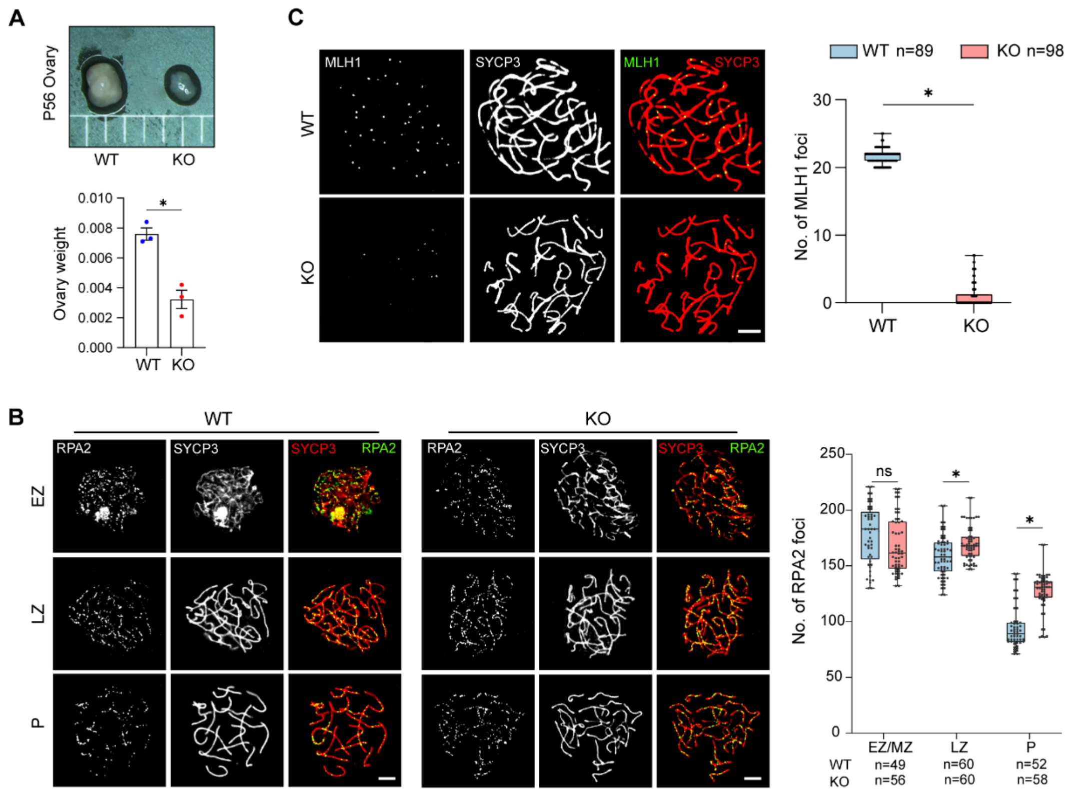

Fig. S6 Meiotic defects in *Zcwpw2* KO female mice. (A) Representative images of ovaries from P56 WT and *Zcwpw2* KO females (left), with quantification of ovary weight relative to body weight (right). P, postnatal day. (n = 3 biologically independent WT mice and KO mice; Two-tailed Student's t test; $*p < 0.05$; error bars, s.e.m.). **(B)** Immunofluorescence staining of RPA2 (green) and SYCP3 (red) in oocytes at early early zygotene (EZ), late zygotene (LZ), and pachytene or pachytene-like (P) stages. Scale bars, 5 µm. n, the number of oocytes. (Two-tailed Student's t test; $*p < 0.05$; ns, no significance; error bars, s.e.m.). **(C)** Immunofluorescence staining of MLH1 (green) and SYCP3 (red) in WT and KO oocytes at the pachytene or pachytene-like stage (left), with accompanying quantification of MLH1 foci (right). Scale bars, 5 µm. n, the number of oocytes. (Two-tailed Student's t test; $*p < 0.05$; error bars, s.e.m.).

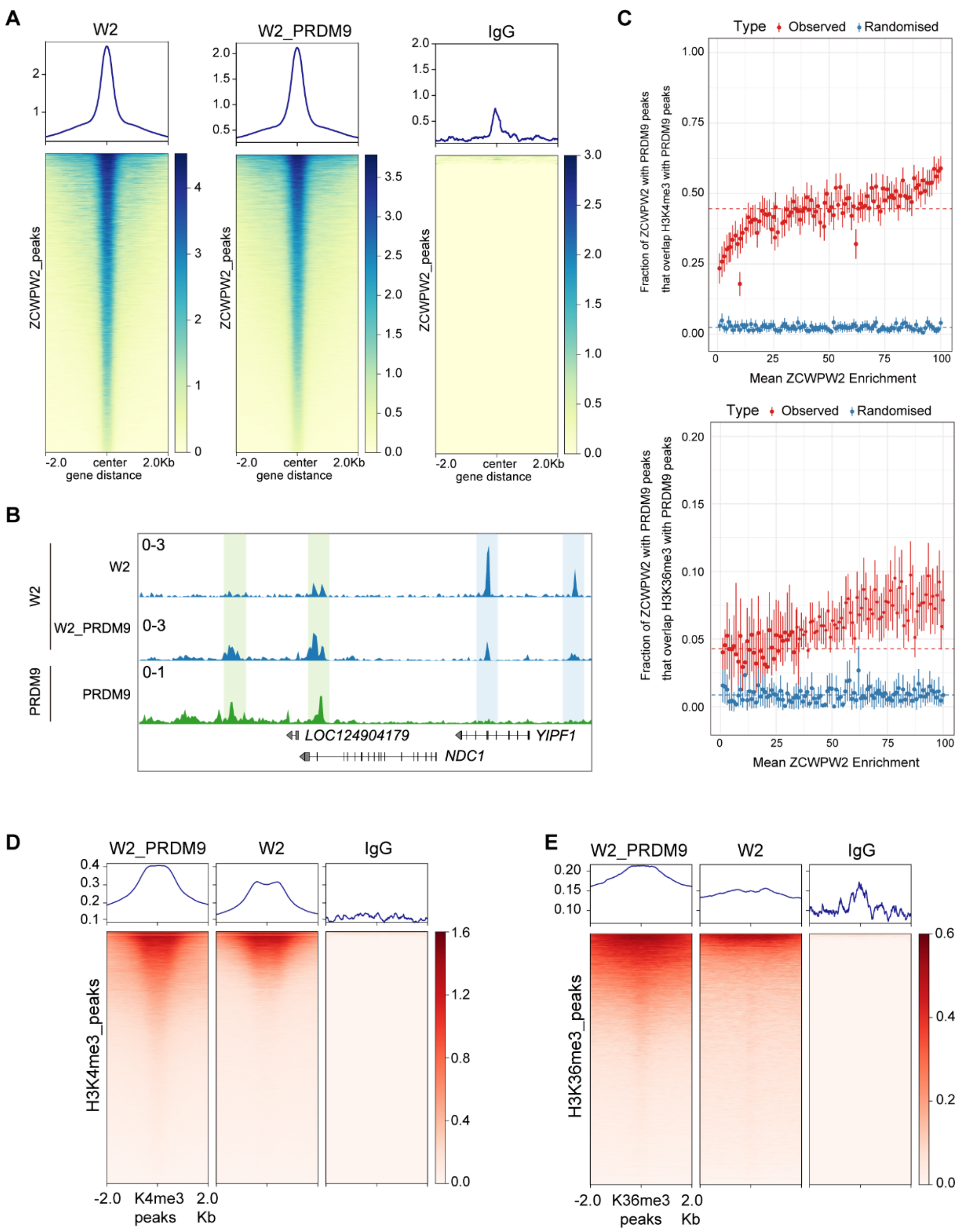

Fig. S7 ZCWPW2 occupancy is enriched at H3K4me3 and H3K36me3 sites in the presence of PRDM9. (A) Heatmaps and signal density plots showing CUT&Tag enrichment of ZCWPW2 at peak regions ±2 kb around the peak center in ZCWPW2 binding center. **(B)** Genome browser view showing ZCWPW2 and PRDM9 CUT&Tag signal tracks at a representative genomic region. **(C)** Scatter plots showing

the fraction of ZCWPW2 peaks overlapping with PRDM9 peaks that are also marked by H3K4me3 (top) or H3K36me3 (bottom), across increasing levels of ZCWPW2 enrichment. Observed values (red) were compared with randomized controls (blue). **(D and E)** Heatmaps and average signal profiles showing the enrichment of ZCWPW2 (W2) at H3K4me3 peaks (D) (±2 kb) or H3K36me3 peaks (E) (±2 kb) in HEK293T cells transfected with Flag-ZCWPW2 alone, co-transfected with Myc-PRDM9, or IgG control.

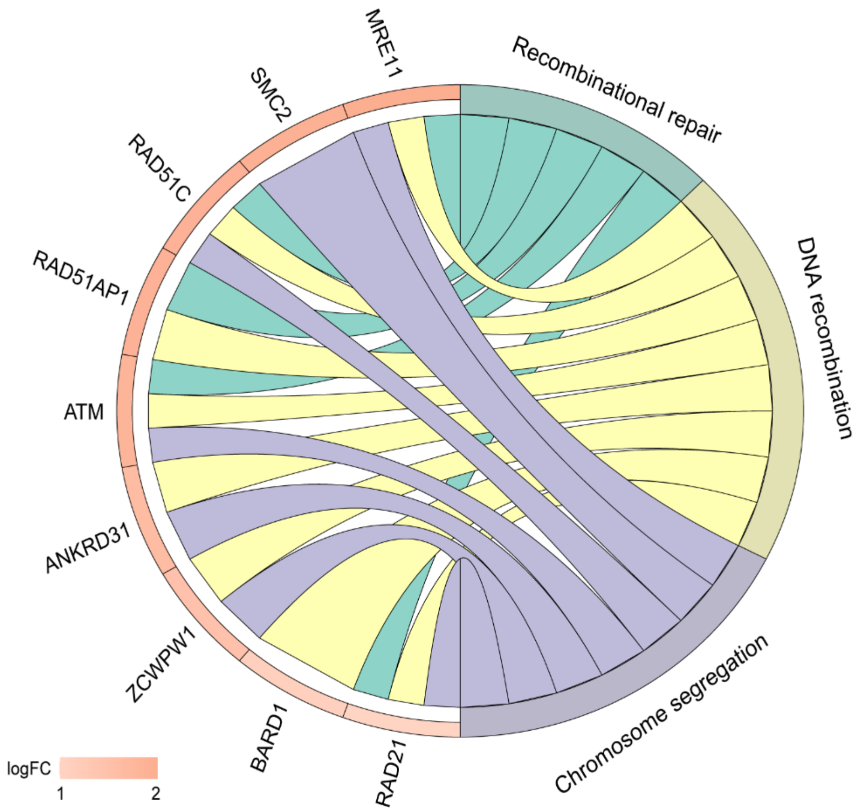

Fig. S8 Meiosis-related genes with ZCWPW2 binding at their promoter regions. Chord diagram illustrating functional associations between genes harboring ZCWPW2 peaks at H3K4me3-marked promoter regions and their enriched GO biological processes.

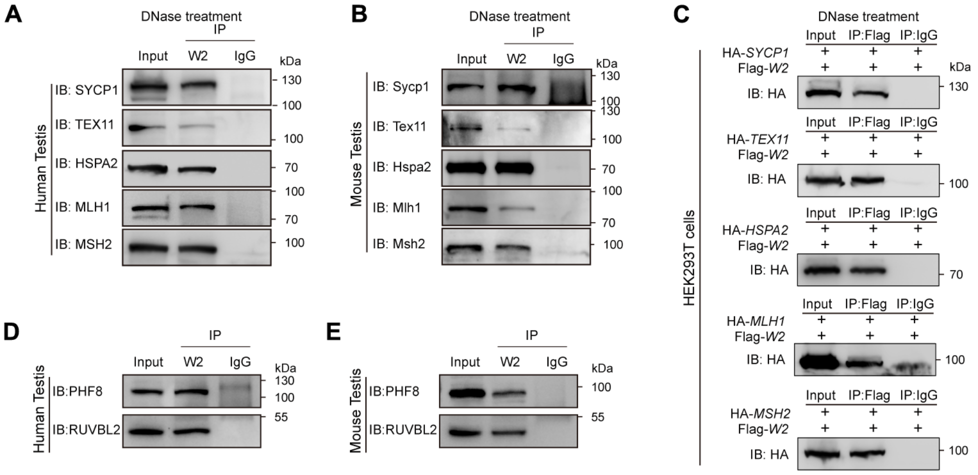

Fig. S9 ZCWPW2 interacts with recombination-associated proteins (under DNase-treated conditions), as well as with the histone demethylase PHF8 and the chromatin-remodeling protein RUVBL2. (A-C) Co-IP assays showing ZCWPW2 interaction with recombination-associated proteins in human (A) and mouse (B) testes and in transfected HEK293T cells (C) under DNase treatment. **(D and E)** Co-IP assays confirmed ZCWPW2 interactions with PHF8 and RUVBL2 in human (D) and mouse (E) testes.

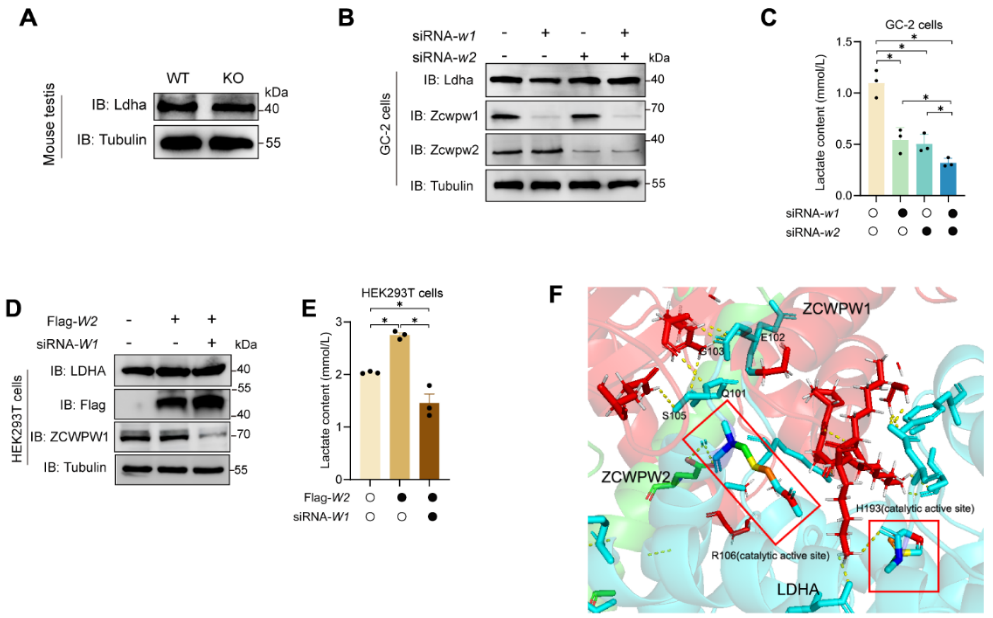

Fig. S10 The ZCWPW1/ZCWPW2 complex enhances LDHA enzymatic stability without changing its expression. (A) Similar levels of Ldha expression were detected in WT and *Zcwpw2* KO testes. **(B and C)** In GC-2 cells, knockdown of Zcwpw1, Zcwpw2, or both did not affect Ldha expression (B), but changes in lactate content were observed in cell lysates under these different transfection conditions (C). (n=3 independent experiment; Two-tailed Student's t test; *p < 0.05; error bars, s.e.m.). **(D and E)** In HEK293T cells, overexpression of ZCWPW2, with or without simultaneous ZCWPW1 knockdown, did not alter LDHA expression (D); however, lactate levels varied among the different transfection conditions (E). (n=3 independent experiment; Two-tailed Student's t test; *p < 0.05; error bars, s.e.m.). **(F)** AlphaFold3 revealed the binding interface between LDHA and the ZCWPW1/ZCWPW2 complex. Among the two known LDHA active sites, residue H193 directly interacts with the complex, whereas residue R106 is positioned adjacent to the binding sites between the complex and LDHA.

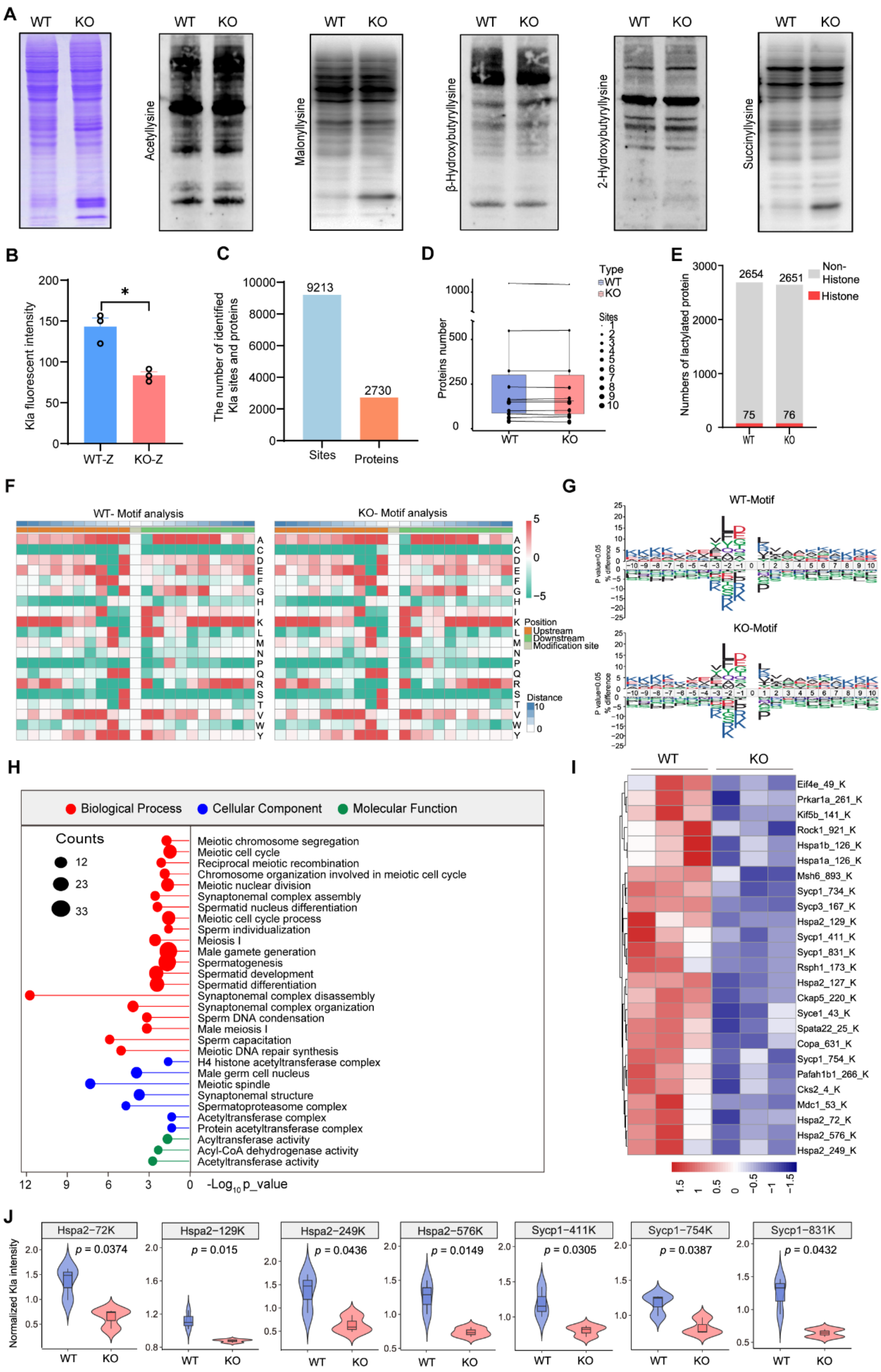
A
WT KO
Acetyllysine
Malonyllysine
β-Hydroxybutyryllysine
2-Hydroxybutyryllysine
Succinyllysine
B
Kla fluorescent intensity
WT-Z KO-Z
*
C
The number of identified Kla sites and proteins
9213
2730
Sites
Proteins
D
Proteins number
Type
WT
KO
Sites
WT KO
E
Numbers of lactylated protein
2654
2651
75
76
Non-Histone
Histone
WT KO
F
WT- Motif analysis
KO- Motif analysis
Position
Upstream
Downstream
Modification site
Distance
G
WT-Motif
KO-Motif
P value=0.05
% difference
H
Biological Process
Cellular Component
Molecular Function
Counts
12
23
33
Meiotic chromosome segregation
Meiotic cell cycle
Reciprocal meiotic recombination
Chromosome organization involved in meiotic cell cycle
Meiotic nuclear division
Synaptonemal complex assembly
Spermatid nucleus differentiation
Meiotic cell cycle process
Sperm individualization
Meiosis I
Male gamete generation
Spermatogenesis
Spermatid development
Spermatid differentiation
Synaptonemal complex disassembly
Synaptonemal complex organization
Sperm DNA condensation
Male meiosis I
Sperm capacitation
Meiotic DNA repair synthesis
H4 histone acetyltransferase complex
Male germ cell nucleus
Meiotic spindle
Synaptonemal structure
Spermatoproteasome complex
Acetyltransferase complex
Protein acetyltransferase complex
Acyltransferase activity
Acyl-CoA dehydrogenase activity
Acetyltransferase activity
-Log10 p_value
I
WT KO
Eif4e_49_K
Prkar1a_261_K
Kif5b_141_K
Rock1_921_K
Hspa1b_126_K
Hspa1a_126_K
Msh6_893_K
Sycp1_734_K
Sycp3_167_K
Hspa2_129_K
Sycp1_411_K
Sycp1_831_K
Rsph1_173_K
Hspa2_127_K
Ckap5_220_K
Syce1_43_K
Spata22_25_K
Copa_631_K
Sycp1_754_K
Pafah1b1_266_K
Cks2_4_K
Mdc1_53_K
Hspa2_72_K
Hspa2_576_K
Hspa2_249_K
J
Normalized Kla intensity
Hspa2−72K p = 0.0374
Hspa2−129K p = 0.015
Hspa2−249K p = 0.0436
Hspa2−576K p = 0.0149
Sycp1−411K p = 0.0305
Sycp1−754K p = 0.0387
Sycp1−831K p = 0.0432
WT KO

Fig. S11 Lactylome in *Zcwpw2* WT and KO testes. (A) Coomassie brilliant blue staining (left) and immunoblot analyses of various post-translational modification (right) in testicular protein lysates from WT and *Zcwpw2* KO mice. **(B)** Bar graph showing the Kla fluorescence intensity in WT and *Zcwpw2* KO zygotene spermatocytes. (n = 3 biologically independent WT mice and KO mice; Two-tailed Student's t test; $*p < 0.05$; error bars, s.e.m.). **(C)** Bar graphs depicting the total number of lactylated proteins and sites identified in testes from WT and *Zcwpw2* KO mice. **(D)** Box plots displaying the number of proteins carrying different quantities of Kla sites in WT and *Zcwpw2* KO testes. Dot size represented the number of Kla sites per protein. **(E)** Bar graphs showing the number of lactylated histone (red) and non-histone (gray) proteins in WT and *Zcwpw2* KO testes. **(F)** Heatmaps depicting amino acid frequencies surrounding Kla sites in WT (left) and *Zcwpw2* KO (right) testes. Alanine (A) and lysine (K) were enriched both upstream and downstream of modification sites, while cysteine (C), serine (S), proline (P), and histidine (H) were underrepresented. **(G)** Sequence logo plots comparing amino acid distribution around Kla sites (positions –10 to +10) in WT (top) and KO (bottom) testes. Motif preferences were nearly identical between WT and KO. **(H)** Dot plot representing significantly enriched GO among downregulated lactylated proteins, categorized into Biological Process (red), Cellular Component (blue), and Molecular Function (green) categories. Dot size indicated the number of proteins annotated to each term. $-\log_{10}$P.value reflected the magnitude of enrichment, where positive values represent enrichment in upregulated proteins and negative values indicate enrichment in

downregulated proteins. **(I)** Heatmap showing hierarchical clustering of significantly downregulated Kla sites between WT and KO testes. **(J)** Violin plots validating significant reductions in Kla levels at specific lysine residues on recombination-related proteins in *Zcwpw2* KO testes. (n = 3 biologically independent WT mice and KO mice; Two-tailed Student's t test)

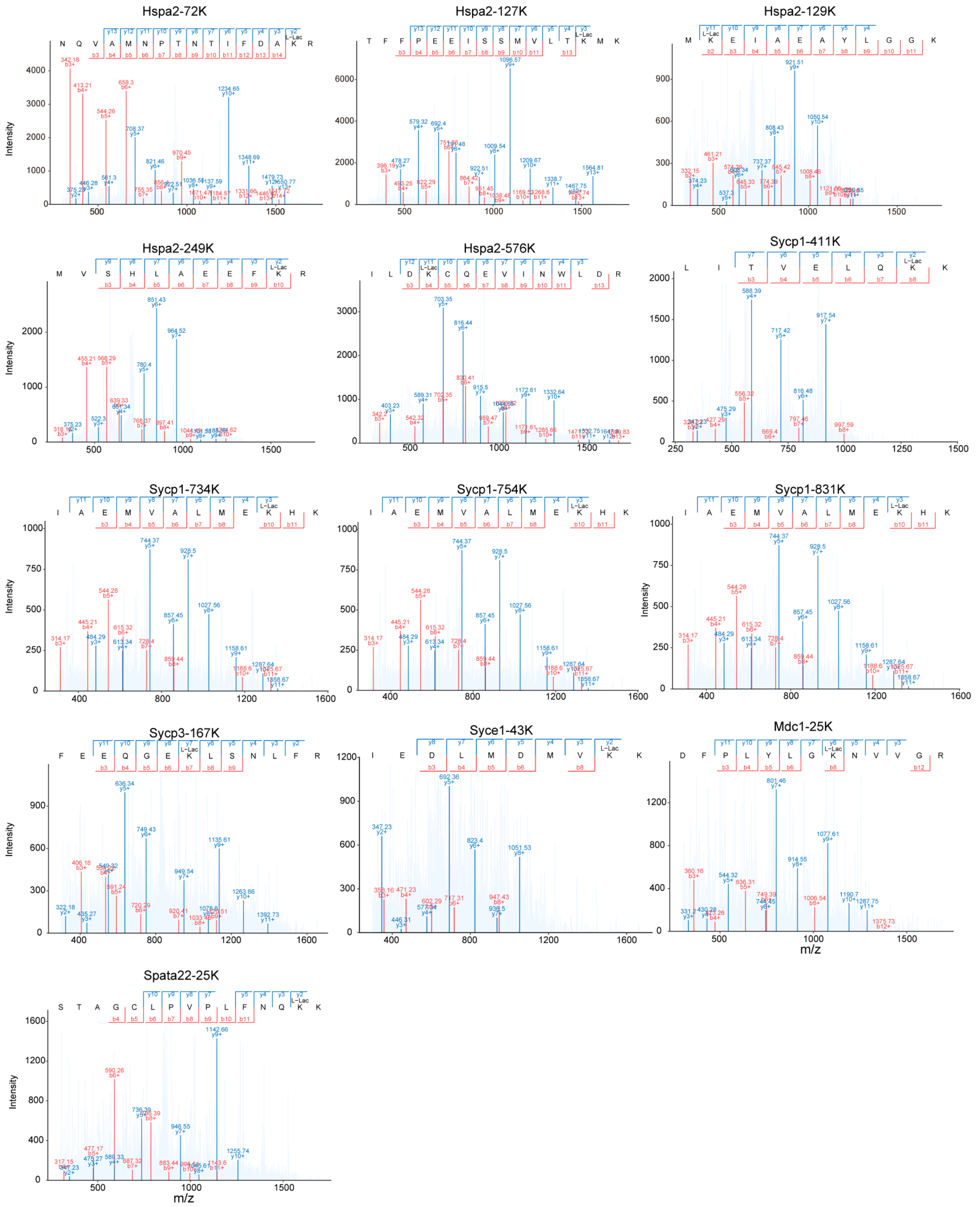

Fig. S12 Mass spectrometry of lactylation at key recombination-related protein sites. Representative LC–MS/MS spectra confirming lysine lactylation (Kla) on meiotic recombination–related proteins. Shown are peptide spectra for Hspa2 (K72, K127, K129, K249, K576), Sycp1 (K411, K734, K754, K831), Sycp3 (K167), Syce1 (K43), Spata22 (K25), and Mdc1 (K25). The x-axis represents mass-to-charge ratio

(m/z) and the y-axis indicates ion intensity. Red peaks correspond to y ions (C-terminal fragments) and blue peaks correspond to b ions (N-terminal fragments). The matched b/y ion series validate the peptide sequences, and the mass shift of +72.02 Da at the indicated lysine residues confirms site-specific lactylation.

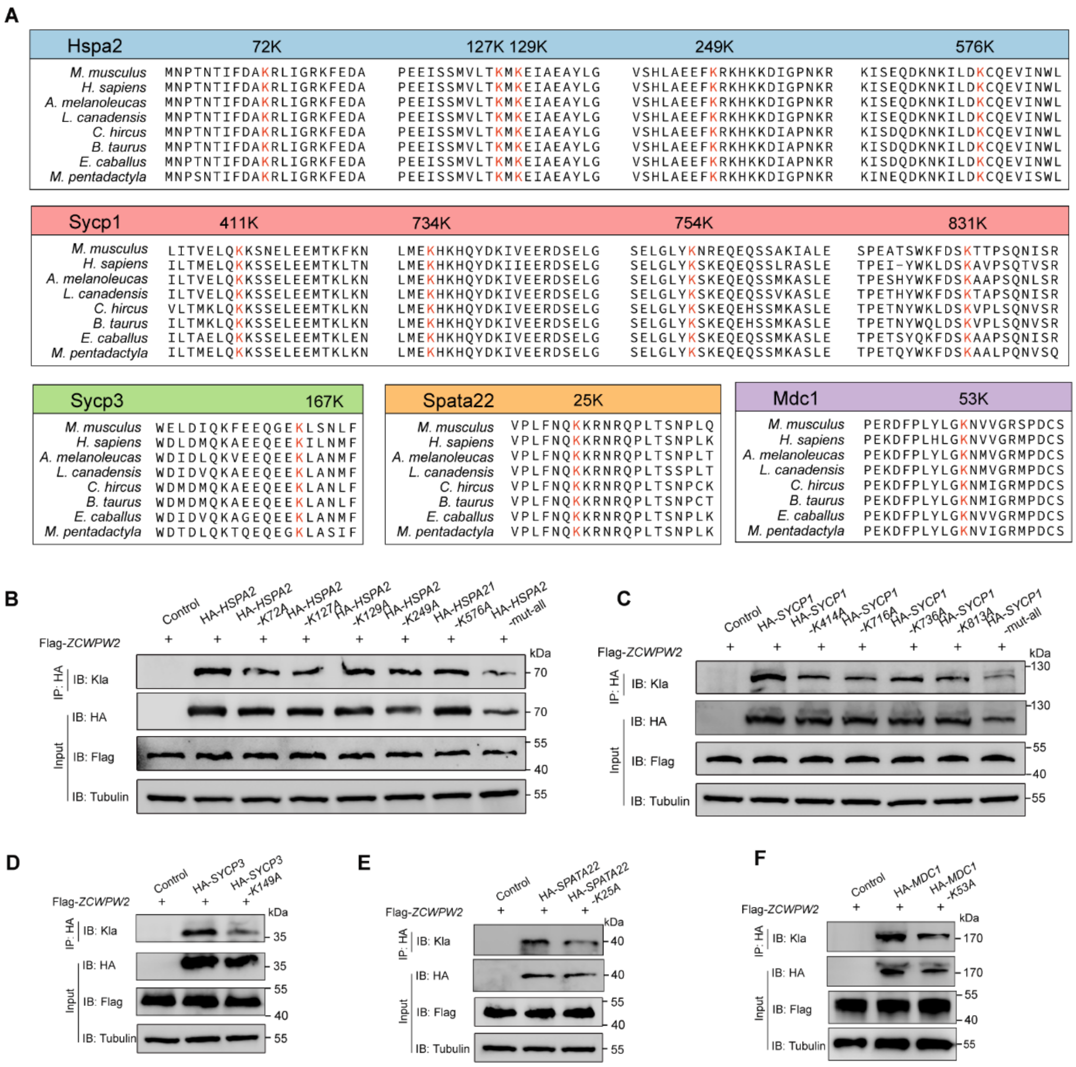

Fig. S13 Conservation and sequence context of ZCWPW2-regulated lactylation sites on meiotic proteins. (A) Multiple sequence alignment of Kla sites on selected recombination-associated proteins (Hspa2, Sycp1, Sycp3, Spata22, and Mdc1) across representative species. Conserved Kla residues are highlighted in red, indicating strong evolutionary conservation. **(B-F)** Co-IP assays showing that single-point (K→A) and multiple-point lysine mutants of HSPA2 (B), SYCP1 (C), SYCP3 (D), SPATA22 (E), and MDC1 (F) exhibit reduced Kla signal.

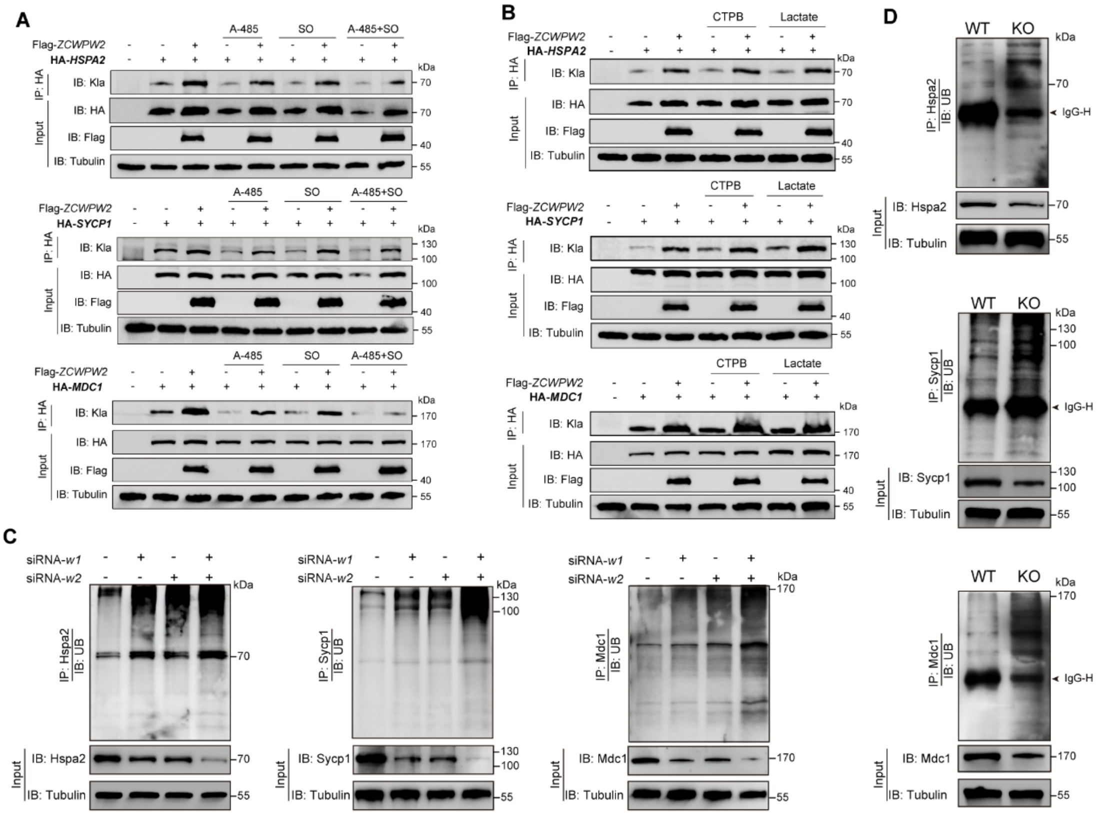

Fig. S14 ZCWPW2 promotes protein lactylation and stabilizes their abundance by inhibiting ubiquitin-mediated degradation. (A) Co-IP assays in HEK293T cells transfected with Flag-*ZCWPW2* and HA-*HSPA2*, *SYCP1*, or *MDC1*, followed by treatment with the EP300 inhibitor A-485, the LDHA inhibitor sodium oxamate (SO), or their combination (A-485+SO). **(B)** Co-IP assays of HEK293T cells co-transfected with Flag-*ZCWPW2* and HA-*HSPA2*, *SYCP1*, or *MDC1*, followed by treatment with the EP300 activator CTPB or lactate. **(C)** In GC-2 cells, knockdown of *Zcwpw1*, *Zcwpw2*, or both led to increased ubiquitination of recombination-associated proteins, accompanied by a reduction in their abundance, with the effect being more pronounced in the double-knockdown group. **(D)** Testes from juvenile *Zcwpw2* KO mice exhibited markedly elevated ubiquitination of recombination-associated proteins compared to WT mice.

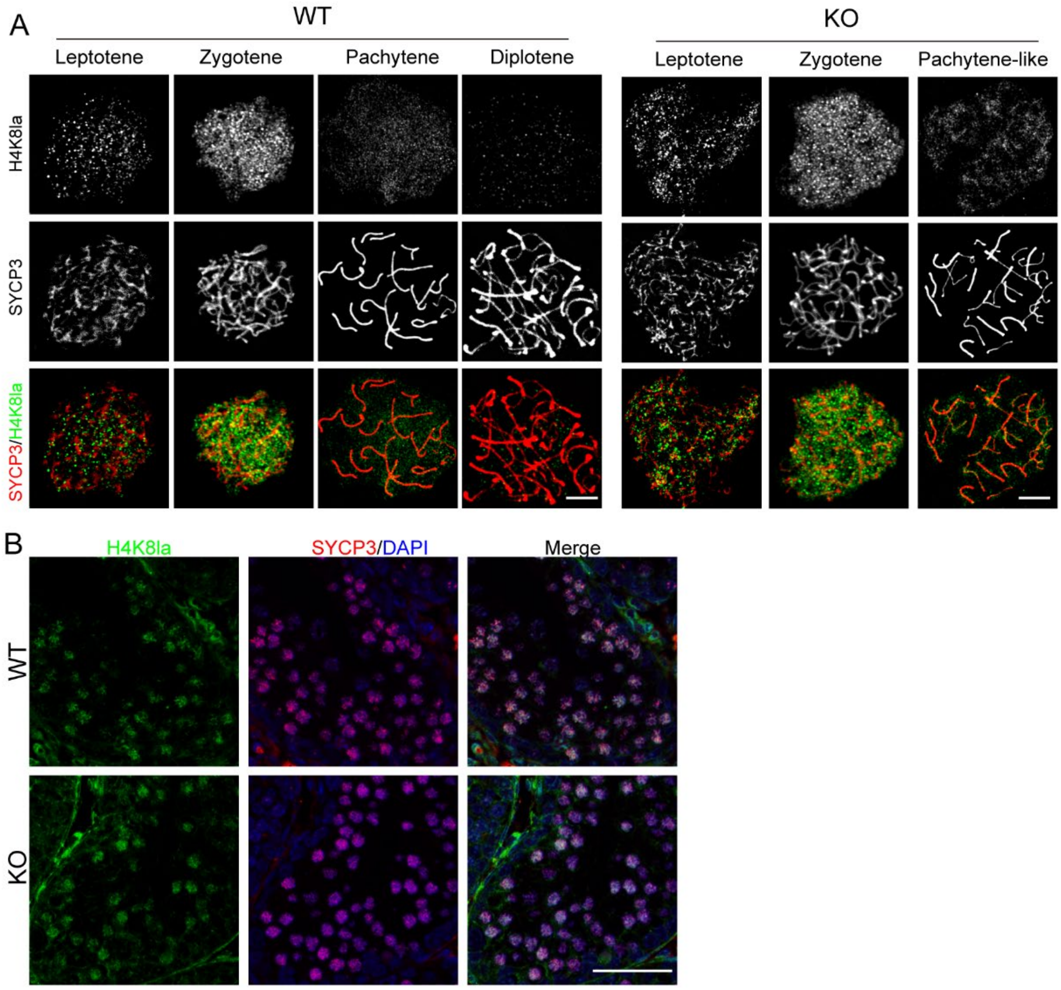

Fig. S15 H4K8la levels are comparable between WT and KO testes. (A) Immunostaining of H4K8la on chromosome spreads showing its distribution in spermatocytes at distinct meiotic stages. Scale bar, 50 μm. (n = 3 biologically independent WT mice and KO mice). **(B)** Representative immunofluorescence staining of H4K8la in cross-sections of juvenile testes from WT and KO mice. Scale bar, 5 μm. (n = 3 biologically independent WT mice and KO mice).

.

Table S1. Comprehensive list of primer sequences employed for RT-PCR in the present study.

Application of primers	Sequences
**Mouse**	
Zcwpw1	F 5' TGGCAGCATTACAGACTCACA 3'<br>R 5' ATGAGCTGGGCTGAGGTTTC 3'
Zcwpw2	F 5' ATGAGAGCTGCCTGAAATGGA 3'<br>R 5' GTAGCTGGGGTCAGTGTTCAT 3'
Prdm9	F 5' CTGGACAGCACTCTGGGAAA 3'<br>R 5' GCAGACTGAGGACTGAGTGG 3'
Rad51c	F 5' AAATGGCCTCGCCCAACAAA 3'<br>R 5' GCCCCCAGCTTTCCCCTAAT 3'
Mre11a	F 5' ATCCGACTACGGGTGGACTA 3'<br>R 5' TCTTGAACTGCTTCGCCCAT 3'
Ankrd31	F 5' TGCTGCCTATTCGTCCTTTCA 3'<br>R 5' CAGGGAAGCTCCTCGTCTTC 3'
Bard1	F 5' TTGTGGGAGCAACATTCCGA 3'<br>R 5' GGTGAAAGGCTAAGGGGGTC 3'
Rad51ap1	F 5' TTACCCCAGGCAGTTGGTCT 3'<br>R 5' CTGGCGCTAATCGGGAGAG 3'
Atm	F 5' GTGTGATTTTTCAGGGGATTTGGA 3'<br>R 5' CCTCCCAGGCCACTCTGTAT 3'
Smc2	F 5' GCCTATCAGTTTTTGCGGGC 3'<br>R 5' GCTCTTTGCGCTTGGTTTCT 3'
Rad21	F 5' CCCCCTCAAGGAGTTAAGCG 3'<br>R 5' CTGCCTGCTTTCGGTTTGTG 3'
Ep300	F 5' GTTGAGTCCGCATCCCTCTC 3'<br>R 5' TGAGAGTTTAGGCCGCTTGG 3'
Hspa2	F 5' TAACCAACGACAAGGGTCGG 3'<br>R 5' GTCTTCCACGGTCTGCTTGA 3'
Mdc1	F 5' TTGGAGAGGGAAATCAGGGGA 3'<br>R 5' AACACCTTCTTTGGCTCTCTGT 3'
Sycp1	F 5' ACCGTTGGACAACGATTGCT 3'<br>R 5' ATCCATTGCAAGTAAAAGCAACA 3'
Actin	F 5' CCTGAGCGCAAGTACTCTGTGT 3'<br>R 5' GCTGATCCACATCTGCTGGAA 3'
**Human**	
Sanger sequencing primer for mutation of *ZCWPW2*	F 5' CCCTGCCATTAGTCTCCTTGAAT 3'<br>R 5' GATAACTAAGCTCAGTGCCCCTTA 3'
RAD51C	F 5' TTGGTGAGTTTCCCGCTGTC 3'<br>R 5' CTGGGTATGCTCCTGCTCAA 3'
MRE11	F 5' TGGAGAAAGATGCAGTCAGAGG 3'<br>R 5' CAGGCCGATCACCCATACAA 3'
ANKRD31	F 5' TGCCCTGGAGAAGGTTGCTG 3'<br>R 5' CTGGCGAAACGACCCAATGAA 3'
BARD1	F 5' TACCATGCGAGACCCGATTC 3'<br>R 5' GGAGCCTTCCAGACTTTGCC 3'
RAD51AP1	F 5' ACAGTCACCACTAATGTGCAG 3'<br>R 5' CCAGGTGCAAAGTCTGGTTC 3'
ATM	F 5' GCTTATCTGCTGCCGTCAA 3'<br>R 5' GGCTTGTGTTGAGGCTGATAC 3'
SMC2	F 5' TGTCTCAGGTTCGGGCTTCT 3'<br>R 5' CCACCAATAACCACCTGCCT 3'
RAD21	F 5' AAACAAGCTGCCGCAAAGTT 3'<br>R 5' TCCAGGTGTTGCGATGATGT 3'
EP300	F 5' CCCTGCCTCCCATTGTTGAT 3'<br>R 5' TCTCCACATGGTGCTTGCAT 3'
HSPA2	F 5' GACGAGAAACTGAGGGGCAA 3'<br>R 5' TCTCTGCCATCTGGTTTCGG 3'
MDC1	F 5' CTTTGCTGACTTGCTCTGCC 3'<br>R 5' AGTCCTCAGCCAACAGAAGC 3'

SYCP1	F 5' TGGGAGGCGATTCCACTTTC 3'<br>R 5' ACAGTCAGAATTACCAACCTGCT 3'

Table S2. Primers for genotyping and sgRNA target sequences.

Application of primers	Sequences
sgRNAs for *Zcwpw2* KO mice	5' TCATCCACGCTGTCGAGTGGGGG 3';
Zcwpw2 KO genotyping	F 5' CTTGCTTACTGTCTTTGAAAGGT 3'<br>R 5' ACCACGTTACCAGTGGAAGAG 3'

Table S3. Comprehensive list of antibodies employed in the study.

Antibody or dye (application and dilution ratio)	Source	Identifier
Rabbit-Anti-ZCWPW2 (IF,1:50)	Bioss	bs-18469R
Rabbit-Anti-ZCWPW2 (WB,1:1000; IP)	Atlas antibodies	HPA035035
Rabbit-Anti-ZCWPW1 (WB, 1:1000)	Abclonal	A7596
Rabbit-Anti-ZCWPW1 (IF, 1:100)	This paper	Gift from Prof. Kui Liu
Mouse-Anti-SYCP3 (IF,1:20)	Santa Cruz Biotechnology	sc-74569
Rabbit-Anti-SYCP3 (IF,1:1000; WB,1:1000)	Abcam	ab934751
Mouse-Anti-SYCP1 (IF,1:50; WB,1:1000; IP)	Novus	NB300-229
Mouse-Anti-gamma H2A.X (IF,1:1000)	Abcam	ab26350
Rabbit-Anti-HORMAD1 (IF,1:100)	Atlas antibodies	HPA028346
Rabbit-Anti-MSH4 (IF, 1:50)	This paper	Gift from Prof. Qinghua Shi
Rabbit-Anti-TEX11 (IF, 1:100; WB,1:1000; IP)	This paper	Gift from Prof. Qinghua Shi
Rabbit-Anti-MLH3 (IF, 1:100)	This paper	Gift from Prof. Mengcheng Luo
Mouse-Anti-MLH1 (IF,1:100)	Cell signaling technology	3515
Rabbit-Anti- RPA2 (1:50)	Abcam	ab76420
Rabbit-Anti-RAD51(IF,1:200)	Millipore	PC130
Rabbit-Anti-SYCE1 (IF,1:100; WB, 1:1000; IP)	Invitrogen	PA5-77174
Rabbit-Anti-MLH1 (WB,1:1000; IP)	Abcam	ab92312
Rabbit-Anti-MSH2 (WB,1:1000; IP)	Cell signaling technology	2017
Rabbit-Anti-HSPA2 (IF,1:100; WB, 1:1000; IP)	Atlas antibodies	HPA000798
Rabbit-Anti- LDHA (WB, 1:1000; IP)	Novus	NBP1-48336
Rabbit-Anti-LDHB (WB, 1:1000; IP)	Invitrogen	PA5-27505

Rabbit-Anti-LDHC (WB, 1:1000; IP)	Abcam	ab222910
Rabbit-Anti-SPATA22 (WB, 1:1000; IP)	Proteintech	16989-1-AP
Rabbit-Anti-MDC1 (IF,1:50; WB, 1:1000; IP)	Novus	NB100-395
Rabbit-Anti-MSH6 (WB, 1:1000; IP)	Abcam	ab92471
Mouse-Anti-Flag (WB, 1:3000; IP)	Proteintech	66008-4-Ig
Rabbit-Anti-HA (WB, 1:3000; IP)	Cell signaling technology	3724
Rabbit-Anti-Ki67 (IF, 1:1000)	Abcam	ab15580
Rabbit-Anti-Alpha Tubulin (WB, 1:3000)	Proteintech	80762-1-RR
Rabbit-Anti-PHF8 (WB, 1:1000; IP)	Cell Signaling Technology	93801S
Rabbit-Anti-RUVBL2 (WB, 1:1000; IP)	Proteintech	10195-1-AP
Rabbit-Anti-L-Lactyllysine (WB, 1:100; IP)	PTM BIO	PTM-1401RM
Rabbit-Anti-Malonyllysine (WB, 1:1000)	PTM BIO	PTM-901
Rabbit-Anti-β-Hydroxybutyryllysine (WB, 1:1000)	PTM BIO	PTM-1201RM
Rabbit-Anti Ubiquitin (WB, 1:1000)	Cell signaling technology	58395
Acylation Antibody Sampler Kit (WB, 1:1000)	PTM BIO	PTM-6681

Table S4. Primers for siRNA design and plasmid construction.

Application of primers	Sequences
siRNAs sequences of *ZCWPW1*	5' GGAGCCAGGAATTACTGCTTCTGCT 3'
siRNAs sequences of *Zcwpw1*	5' GAACTCTTACCAGTGCAGAATTTGA 3'
siRNAs sequences of *Zcwpw2*	5' GGAATTGGCCAAGTTGGCCAGGAAT 3'
HA-*ZCWPW1-Δzf-CW* plasmid	F 5' AATGTCTGACCTGGACAGGGCTTGAGAGTGATG 3'<br>R 5' CTGTCCAGGTCAGACATTGACCAAAACCACTTATCTCTCCTTTTTGC 3'
HA-*ZCWPW1-ΔPWWP* plasmid	F 5' GAGAGTGATTGGAGCCGATTCAACGGATCTAAC 3'<br>R 5' ATCGGCTCCAATCACTCTCAAGCCCTGTCCAGG 3'
Flag-*ZCWPW2-Δzf-CW* plasmid	F 5' GTAAACAAATTCCCTGAAGAGTCTCAGCTTCATCAGT 3'<br>R 5' CTTCAGGGAATTTGTTTACATACATGTTTTCCACTGAGGA 3
Flag-*ZCWPW2-ΔPWWP* plasmid	F 5' TCAGTGTATGTGCTGCCTATCAAAACTACAAGATAAATCCGAAACACATG 3'<br>R 5' GCAGCACATACACTGATGAAGCTGAGACTCTTCAGG 3'
HA-*HSPA2-K72A* plasmid	F 5' ACACCATCTTCGACGCCGCGAGGCTGATTGGACG 3'<br>R 5' GGCGTCGAAGATGGTGTTGGTGGGG 3'
HA-*HSPA2-K127A* plasmid	F 5' ATCCTCCATGGTCCTCACGGCGATGAAGGAGATCGC 3'<br>R 5' CGTGAGGACCATGGAGGATATCTCCTC 3'
HA-*HSPA2-K129A* plasmid	F 5' GTCCTCACGAAGATGGCGGAGATCGCGG 3'<br>R 5' CATCTTCGTGAGGACCATGGAGGATATCTCCTC 3'
HA-*HSPA2-K249A* plasmid	F 5' CTGGCGGAGGAGTTCGCGCGCAAGCACA 3'<br>R 5' GAACTCCTCCGCCAGGTGGCTCACC 3'
HA-*HSPA2-K576A* plasmid	F 5' AAAAACAAGATCCTCGACGCGTGTCAGGAGGTGATC 3'<br>R 5' GTCGAGGATCTTGTTTTTGTCCTGC 3'
HA-*SYCP1-K414A* plasmid	F 5' TACTTACCATGGAGCTTCAAGCGAAATCAAGTGAGC 3'<br>R 5' TTGAAGCTCCATGGTAAGTATTTTCAATTG 3'
HA-*SYCP1-K716A* plasmid	F 5' GAAATGGTAGCACTTATGGAAGCACATAAGCACCAAT 3'<br>R 5' TTCCATAAGTGCTACCATTTCAGCTATTTT 3'
HA-*SYCP1-K736A* plasmid	F 5' AGAGACTCAGAATTAGGACTTTATGCGAGCAAAGAACAAG 3'<br>R 5' ATAAAGTCCTAATTCTGAGTCTCTTTCTTCAATG 3'
HA-*SYCP1-K813A* plasmid	F 5' TATTGGAAATTGGATTCTGCAGCAGTTCCTTCACA 3'<br>R 5' AGAATCCAATTTCCAATAAATTTCAGGT 3'

HA-*SPATA22-K25A* plasmid	F 5' CCGTTGTTCAATCAGGCAAAGAGGAACA 3'<br>R 5' CTGATTGAACAACGGAACAGGCAAA 3'
HA-*SYCP3-K149A* plasmid	F 5' AAGCTGAGGAACAAGAAGAAGCAATACTTAATATGTTTCGAC 3'<br>R 5' TTCTTCTTGTTCCTCAGCTTTCTGCATATC 3'
HA-*MDC1-K53A* plasmid	F 5' CCACTACACCTCGGGGCGAATGTGGTAGGC 3'<br>R 5' CCCGAGGTGTAGTGGGAAATCTTTT 3'
HA-*MSH6-K896A* plasmid	F 5' GTCATCTCTCTGCAGACAGCAAATCCTGAAGGTC 3'<br>R 5' TGTCTGCAGAGAGATGACCTGCTTA 3'
Myc-*LDHA-R106A* plasmid	F 5' ACGTCAGCAAGAGGGAGAAAGCGCTCTTAATTTGGTC 3'<br>R 5' GCTTTCTCCCTCTTGCTGACGTGCCCCA 3'
Myc-*LDHA-H193A* plasmid	F 5' GGGTGGGTCCTTGGGGAAGCTGGAGATTCCAGTGT 3'<br>R 5' TTCCCCAAGGACCCACCCATGACAGCTT 3'
RAD51AP1 promoter plasmid	F 5' AGAACATTTCTCTATCGATAATCAGGAATGTTGAAAAAAAAAAG 3'<br>R 5' ATGTTTTTGGCGTCTTCCATTGCCTATTTCTCTGGCCTAAT 3'
ZCWPW1 promoter plasmid	F 5' AGAACATTTCTCTATCGATAGCCAGTCGGGGTAACGAGG 3'<br>R 5' ATGTTTTTGGCGTCTTCCATCTGCTGGTCTCCTTCACGCC 3'
BARD1 promoter plasmid	F 5' AGAACATTTCTCTATCGATACCTGAATCCAACTGAAACGGTAGTT 3'<br>R 5' ATGTTTTTGGCGTCTTCCATATCATTTATTCTATCACAACCACCAAACTC 3'
RAD51C promoter plasmid	F 5' AGAACATTTCTCTATCGATATGCTCGGGATACGACTCCC 3'<br>R 5' ATGTTTTTGGCGTCTTCCATCTTGGGGGACACAGTCTGC 3'
MRE11 promoter plasmid	F 5' AGAACATTTCTCTATCGATAGCACAAAGCAGTGCAGATGTGCTGAACTCAAGGACCTAGGGGCGAC 3'<br>R 5' ATGTTTTTGGCGTCTTCCATAAATTCTAAAAACAAAATTACATCATAAAACACATTTTTTAAAAGCCTGCACGTATTTAACCAA 3'
ATM promoter plasmid	F 5' AGAACATTTCTCTATCGATAGTAAGCTGGGAGGCAAAACCCCAAAGCTTCCCTACCA 3'<br>R 5' ATGTTTTTGGCGTCTTCCATCGCACACGACTGAATTTCCCAGC 3'
ANKRD31 promoter plasmid	F 5' AGAACATTTCTCTATCGATAAAGATAATCACCAATCAAGCATTTTAAATAGCAGTGGCTTGA 3'<br>R 5' ATGTTTTTGGCGTCTTCCATGGGCAGCTCCTTTTCTTCCAGGTCGCTCTCCGTC 3'
SMC2 promoter plasmid	F 5' AGAACATTTCTCTATCGATATGCTATCCACTAAAGAGAAAGCACCCC 3'<br>R 5'

	ATGTTTTTGGCGTCTTCCATCTACAACACCTGCCACACCGCTCTCAACACCCG 3'
RAD21 promoter plasmid	F 5' AGAACATTTCTCTATCGATAAATATTCACCGCACTGGAAAGC 3'<br>R 5' ATGTTTTTGGCGTCTTCCATATTTAAGAAAATAGAAATTTTTGCCTGAAAACCAAT 3'
EP300 promoter plasmid	F 5' AGAACATTTCTCTATCGATACCTGCGCCGCCGCCGGAGCGGGGCCTCCTCGGTGGGCTCC 3'<br>R 5' ATGTTTTTGGCGTCTTCCATCTGCAAACATATTACAAACTGCAGAA 3'

Table S5. A comprehensive list of primer sequences employed for PCR of the promoter in the present study.

Application of primers	Sequences
ATM promoter	F 5' CAGTCACGCAGGGTTTGAAC 3'<br>R 5' ACTGAATTTCCCAGCGCAGA 3'
RAD51AP1 promoter	F 5' CGAAAGTGCAGGGACAAAGC 3'<br>R 5' GAACTACCCACCTCACAGGC 3'
BARD1 promoter	F 5' CGTCTGGAGGTGTGGCTAAA 3'<br>R 5' GAAAGCAGCGGGGTAATCCA 3'
ZCWPW1 promoter	F 5' GAATTGGGGAAAAATAAAAAGCACT 3'<br>R 5' CTCAGCCGTTTCGGGG 3'
RAD51C promoter	F 5' ACAGCGTGAAAGAGCTCCTC 3'<br>R 5' CAGGATGCAGTGGTTCCCAT 3'
MRE11 promoter	F 5' AGAGGCAAGTTCAGACCGTG 3'<br>R 5' TCGGGATTCCAAGCCTCAAC 3'
ANKRD31 promoter	F 5' TGAATGTGAGGCAAAGATGG 3'<br>R 5' TTTTCTTCCAGGTCGCTCTC 3'
SMC2 promoter	F 5' CCGACGTGGAAACTTCAGAT 3'<br>R 5' TCTAGTTCGCGTTAGGCACA 3'
RAD21 promoter	F 5' AACCCTGGGGGATTTACTTG 3'<br>R 5' AAACACAAGCCTTTCCCAGA 3'
EP300 promoter	F 5' CTCTAGAGCCGCGAGTTCTC 3'<br>R 5' GGAGGGCACAAGAGCACAAA 3'

Table S8. Overlapping promoter-region peaks between ZCWPW2 and histone-modifying enzymes or chromatin remodelers.

Target	Promoter peaks overlapping with ZCWPW2	GEO accession
PHF8	5318	GSE20753
SMARCC1	40	GSE193713
DOT1L	396	GSE196476
PARP1	0	GSE206022
BRD1	1234	GSE244027
KDM1A	1693	GSE223280
SMARCA5	219	GSE281297
KAT7	212	GSE133516
RUVBL2	5539	GSE160738
